# iPSC-RPE patch restores photoreceptors and regenerates choriocapillaris in a pig retinal degeneration model

**DOI:** 10.1172/jci.insight.179246

**Published:** 2025-05-22

**Authors:** Rohan Gupta, Irina Bunea, Bruno Alvisio, Francesca Barone, Rishabh Gupta, Dara Baker, Haohua Qian, Elena Daniele, Casey G. Contreary, Jair Montford, Ruchi Sharma, Arvydas Maminishkis, Mandeep S. Singh, Maria Teresa Magone De Quadros Costa, Amir H. Kashani, Juan Amaral, Kapil Bharti

**Affiliations:** 1 Ocular and Stem Cell Translational Research Section, National Eye Institute, NIH, Bethesda, Maryland, USA.; 2 BioTeam, Life Sciences IT Consulting, Middleton, Massachusetts, USA.; 3 Visual Function Core and; 4 Translational Research Core, National Eye Institute, NIH, Bethesda, Maryland, USA.; 5 Wilmer Eye Institute and; 6 Department of Genetic Medicine, Johns Hopkins University, Baltimore, Maryland, USA.; 7 Consult Services Section, National Eye Institute, NIH, Bethesda, Maryland, USA.

**Keywords:** Ophthalmology, Transplantation, Medical devices, Organ transplantation, iPS cells

## Abstract

Dry age-related macular degeneration (AMD) is a leading cause of untreatable vision loss. In advanced cases, retinal pigment epithelium (RPE) cell loss occurs alongside photoreceptor and choriocapillaris degeneration. We hypothesized that an RPE-patch would mitigate photoreceptor and choriocapillaris degeneration to restore vision. An induced pluripotent stem cell–derived RPE (iRPE) patch was developed using a clinically compatible manufacturing process by maturing iRPE cells on a biodegradable poly(lactic-co-glycolic acid) (PLGA) scaffold. To compare outcomes, we developed a surgical procedure for immediate sequential delivery of PLGA-iRPE and/or PLGA-only patches in the subretinal space of a pig model of laser-induced outer retinal degeneration. Deep learning algorithm-based optical coherence tomography (OCT) image segmentation verified preservation of the photoreceptors over the areas of PLGA-iRPE–transplanted retina and not in laser-injured or PLGA-only–transplanted retina. Adaptive optics imaging of individual cone photoreceptors further supported this finding. OCT-angiography revealed choriocapillaris regeneration in PLGA-iRPE– and not in PLGA-only–transplanted retinas. Our data, obtained using clinically relevant techniques, verified that PLGA-iRPE supports photoreceptor survival and regenerates choriocapillaris in a laser-injured pig retina. Sequential delivery of two 8 mm^2^ transplants allows for testing of surgical feasibility and safety of the double dose. This work allows one surgery to treat larger and noncontiguous retinal degeneration areas.

## Abstract 2

Replacement RPE-patch using iPSC-derived iRPE cells on a PLGA scaffold was tested in a pig model of laser-induced retinal degeneration, showing photoreceptor preservation and choriocapillaris regeneration.

## Introduction

Degenerative diseases of the retina are a major cause of untreatable blindness and vision loss affecting over 300 million individuals worldwide (1, 2). Recent advances in gene therapies are leading to the development of potentially curative options (3, 4). One such example is LUXTURNA (voretigene neparvovec-rzyl) used for the treatment of Leber’s congenital amaurosis, a monogenic disease caused by autosomal recessive mutations in gene RPE65 (5). Gene therapies are, however, often limited to a relatively early disease stage when the relevant cell types are still intact. Furthermore, gene therapies do not provide optimal treatment options for polygenic diseases, such as age-related macular degeneration (AMD) (5, 6). In contrast with gene therapies, cell-based therapies provide the possibility of replacing damaged and degenerated tissues and may work even at late stages of disease. This claim is supported by previous clinical studies where autologous retinal pigment epithelium (RPE) and retinal grafts have been shown to stop disease progression and, in some cases, restore vision (7, 8).

Pluripotent stem cell–derived RPE transplants have been tested in early-stage clinical trials for AMD and RPE-associated monogenic diseases and have provided promising safety results (9, 10). Two different versions of RPE transplants are being tested in patients, both delivered in the subretinal space: (i) RPE cells injected in a cell suspension and (ii) RPE cells transplanted as a monolayer patch on a nondegradable, biodegradable, or biological scaffold (8). The scaffold approach allows delivery of a fully polarized monolayer of RPE and potentially offers a permanent replacement tissue for the degenerated host RPE, whereas cells in the former category need to self-organize into a monolayer structure under the retina (10–12). The monolayer patches range in size from 8 to 16 mm^2^, covering anywhere between 50% and 100% of the macular region (11). The relatively large size of such transplants poses a challenge to test in an appropriate preclinical animal model. For instance, studies performed in rats are often limited to testing transplant pieces smaller than 0.5 mm in diameter that do not provide an appropriate assessment of the human clinical dose (8 or 16 mm^2^) (10) or the human surgical procedure (13) and do not accurately mimic human retinal anatomy (14). Recently, several groups have used pigs as a model to test a large RPE-patch on a scaffold (10, 15). Although most of these studies are limited by the feasibility of the delivery procedure, the safety and efficacy of the RPE-patch was established in some cases (10). An advantage of using pigs for such an assessment is that the pig eye size and anatomy are similar to the human eye, allowing testing of surgical techniques developed for the delivery of the RPE-patch in humans (16, 17). Additionally, pig eyes are compatible with testing clinically relevant techniques used for evaluating human eyes, including color fundus photography, optical coherence tomography (OCT), adaptive optics (AO), fluorescein angiography (FA), OCT-angiography (OCT-A), and electroretinography. OCT-A is a particularly interesting modality, given its extensive use in evaluating choriocapillaris perfusion and degeneration in the eyes of patients with AMD (18). However, there is insufficient evidence supporting the ability of RPE transplants to restore choriocapillaris perfusion in either animal models or patients with AMD. Furthermore, there is also a need for quantitative comparative analyses of RPE transplant versus sham transplant in porcine or other preclinical animal models (19).

In this manuscript, we have addressed these shortcomings of preclinical studies by developing a surgical procedure to deliver more than 1 immediately sequential transplant per eye. This allows evaluation of both the test article [poly(lactic-co-glycolic acid)–induced (PLGA-induced) pluripotent stem cell–derived RPE (iRPE)] and the sham (PLGA-only) transplant in the same pig eye. The host RPE layer of the pig eye was denuded using our previously developed laser procedure (19). Furthermore, we developed quantitative tools to perform a comparative efficacy assessment of the sham and the test transplant measured using clinically relevant modalities OCT, OCT-A, and AO. This work provides a basis to probe mechanistic analysis of RPE and photoreceptor-based cell therapies in preclinical studies.

## Results

### Single-procedure subretinal delivery of PLGA-iRPE and PLGA-only transplants.

**Figure 1:**
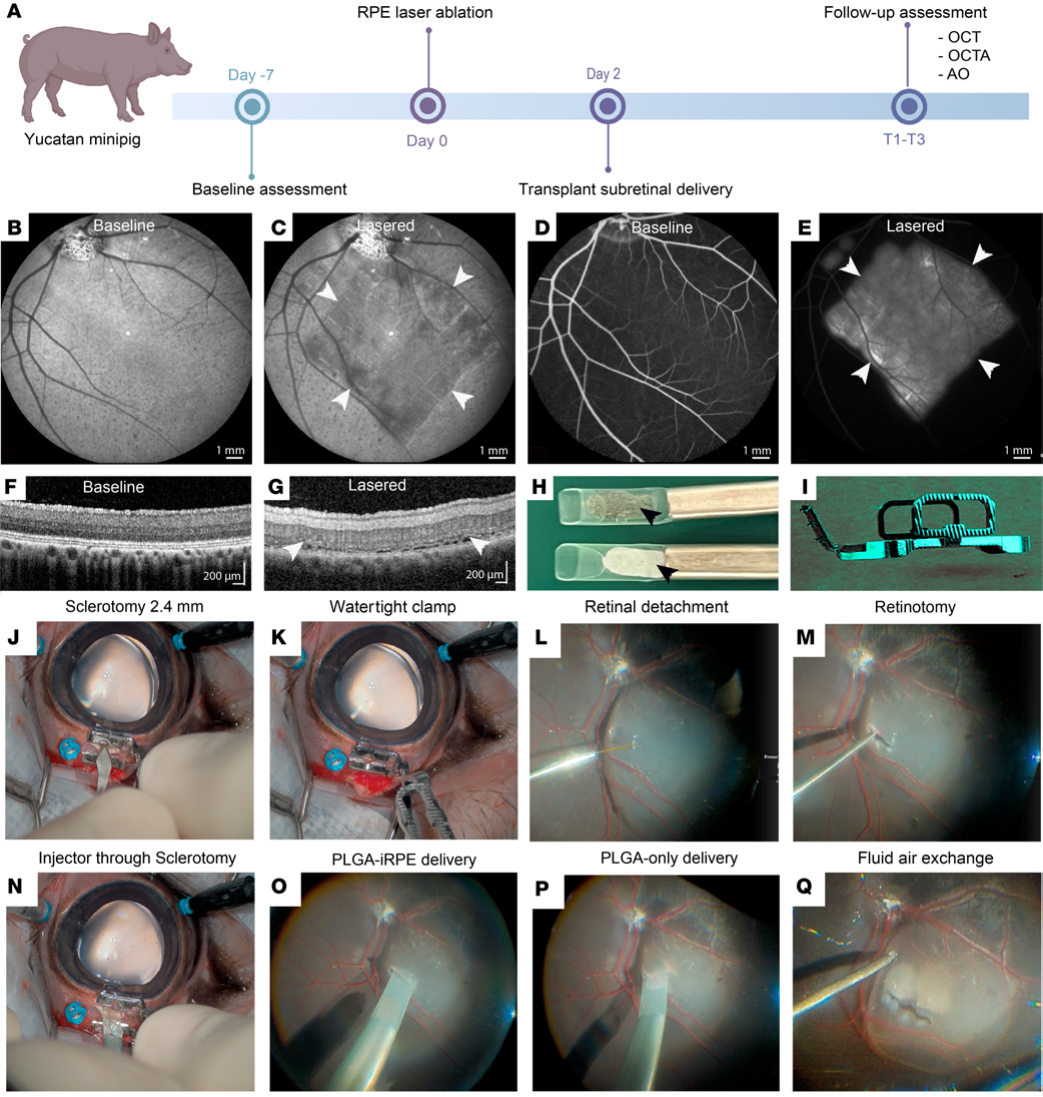
Study and surgery design. (**A**) Animals were evaluated 7 days before surgery (day –7) using optical coherence tomography (OCT), OCT-angiography (OCT-A), and adaptive optics (AO). On the day of laser treatment (day 0), a 7 mm × 7 mm area within the visual streak was ablated with a micropulse laser. Two days after the laser injury, transplants were delivered into the subretinal space using standard vitreoretinal techniques. Animals were followed for up to 80 days. (**B**–**G**) Baseline (**B**, **D**, and **F**) and after laser injury (**C**, **E**, and **G**) fundus photographs (**B** and **C**), FA (**D** and **E**), and OCT (**F** and **G**) of the pig eye. Arrowheads in **C**, **E**, and **G** mark borders of the laser lesion. (**H**) Cannula tips containing PLGA only (bottom) and PLGA-iRPE (top) are marked by arrowheads. (**I**) Scleral clamp was used to secure the sclerotomy. (**J**–**Q**) Surgical technique for immediate sequential delivery of 2 transplants into the subretinal space: After vitrectomy, a 2.4 mm sclerotomy was created using a surgical knife (**J**); sclerotomy was sealed watertight using a clamp (**K**); retinal detachment was created by injecting 0.25% Healon BSS+ solution in the subretinal space (**L**); a 2.5–3 mm retinotomy was created using retinal scissors (**M**); the first cannula was introduced through the clamp (**N**) and transplant delivered subretinally (**O**); after withdrawal of the first cannula and wound securing with the clamp, the second cannula was introduced and transplant delivered (**P**); and retinal detachment was flattened using fluid-air exchange (**Q**).

A previously developed Yucatan pig model with laser ablation of the RPE was used to perform a comparative analysis of PLGA-iRPE and PLGA-only transplants delivered immediately sequentially and placed side by side in the subretinal space (Figure 1A) (10, 19). A 1% duty cycle micropulse laser (1,200–1,800 mW) was used to create RPE and choriocapillaris ablation in an area approximately 50 mm^2^ in size (Figure 1, B–G). Fundus imaging performed immediately after the procedure verified an approximately 50 mm^2^ hypopigmented region suggesting loss of RPE monolayer (Figure 1, B and C). Late-phase (10 minutes) FA revealed leaking dye from choriocapillaris, further verifying disruption of the outer blood-retinal barrier (Figure 1, D and E). OCT validated targeted damage to the RPE and the ellipsoid zone (EZ) region and thickening/swelling of the inner retina (Figure 1, F and G). This image analysis verified the consistency of the laser injury, consistent with published work (19).

A previously developed clinical manufacturing process was used to produce the PLGA-iRPE transplant or the PLGA-only transplant (10). The surgical technique Sharma et al. originally described was modified using a customized wound clamp to control the intraocular pressure during the insertion of 2 cannulas in the eye, one containing a PLGA-only and the other containing a PLGA-iRPE transplant (Figure 1H). The clamp manufactured from surgical stainless steel contains 2 spring-loaded serrated plates. Serrated plates temporarily close the edges of the wound in the sclera using spring force (Figure 1I). A complete core vitrectomy and peripheral vitreous shaving were performed, including induction of a posterior vitreous detachment. Intravitreal triamcinolone acetonide 40 mg/mL (Kenalog) was used to visualize the vitreous and aid in its complete removal. Transplants were delivered into the subretinal space using an approximately 2.4 mm sclerotomy and initial 2.5–3 mm retinotomy (Figure 1, J–Q, and Supplemental Video 1; supplemental material available online with this article; https://doi.org/10.1172/jci.insight.179246DS1). The clamp allowed the successful immediate sequential delivery of 2 transplants and avoided sustained intraoperative hypotony. In cases where watertight sclerotomy clamping was not achieved, the turbulence of the infusion fluid typically impeded the sequential release of the second transplant, and the surgery was terminated after delivery of 1 transplant. This led to a discrepancy in the number of delivered PLGA-only and PLGA-iRPE transplants in our surgical evaluation (Table 1).

Anatomical and functional evaluation was initiated approximately 2 weeks after the surgery, allowing the long-term gas (15% C3F8) to dissipate and the eye to heal from surgical trauma. Postsurgical follow-up was performed using 3 clinically relevant modalities: OCT to monitor retinal layer thickness and morphology, AO to analyze cone photoreceptor cell density at single-cell resolution, and OCT-A to monitor choriocapillaris perfusion (Figure 1A). To avoid animal and operator fatigue, 3 windows were designed for functional assessment: T1 — days 12–28, T2 — days 29–45, and T3 — days 46–80 (Table 1). Our laser-induced RPE ablation pig model combined with a customized clamp allowed for the evaluation and comparison of the 2 transplants in the same subretinal location.

### Qualitative and quantitative OCT analysis shows efficacy of PLGA-iRPE patch.

**Figure 2:**
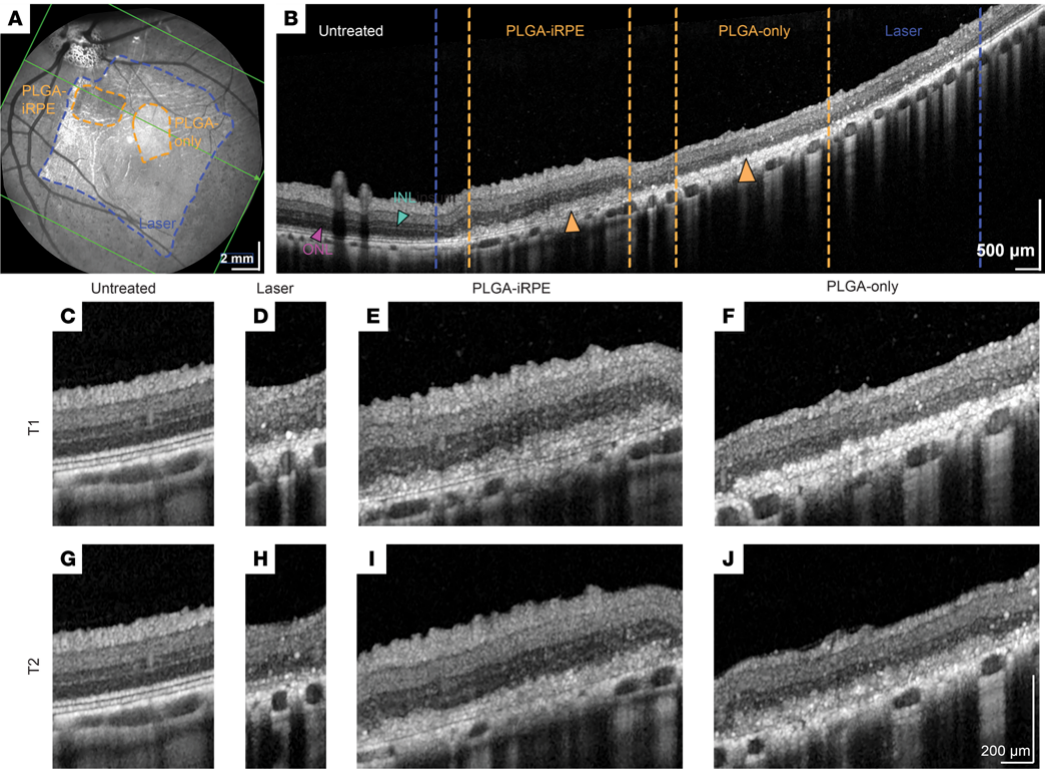
OCT evaluation of PLGA-iRPE and PLGA-only transplants in laser-injured porcine retinas. (**A**) Fundus infrared image of a pig eye at T1, with lasered region marked with blue lines, transplant boundaries marked with orange dotted lines, and the OCT scan position overlaid with the green line. (**B**) OCT B-scan of a pig retina at T1 showing the entire retinal region. Lasered region boundaries are marked with blue dotted lines. Transplants are marked with arrowheads, and their borders are delineated with orange dotted lines. INL, inner nuclear layer; ONL, outer nuclear layer. (**C**–**J**) Higher magnification image of OCT B-scan of untreated retina region at longitudinal time points T1 (**C**) and T2 (**G**); lasered region at T1 (**D**) and T2 (**H**); PLGA-iRPE–transplanted region at T1 (**E**) and T2 (**I**); and PLGA-only–transplanted region at T1 (**F**) and T2 (**J**).

The Heidelberg Engineering Eye Explorer 2 (HEYEX 2) software helped correlate the exact retinal location between the fundus image and the OCT B-scan, allowing us to identify the following regions distinctly within OCT volumes: untreated, laser-injured, PLGA-only transplant, and PLGA-iRPE transplant regions (Figure 2, A and B). The location of the laser injury and the 2 transplants was demarcated on the fundus image; PLGA-only transplants often presented a brighter signal as compared with PLGA-iRPE transplants that appeared darker in fundus photographs, suggesting the presence of pigmented iRPE cells (Figure 2A). Differential reflectance of the 2 transplants allowed us to monitor their location at all 3 follow-up time points. Qualitative longitudinal analysis of OCT B-scans in the transplant area further verified the presence of 2 distinct hyper-reflective bands in the outer retina in the lasered area, corresponding to the PLGA-only and PLGA-iRPE transplants (Figure 2, B–J). Higher magnification OCT B-scans at T1 and T2 time points revealed improved preservation of the photoreceptor outer nuclear layer (ONL) thickness in PLGA-iRPE–transplanted retinal regions, as compared with PLGA-only or lasered regions that had a relatively thinner ONL, suggesting a protective effect of iRPE cells on photoreceptor cell number (Figure 2, C–J). Overall, OCT B-scans suggested that PLGA-iRPE protects the retina overlying the laser lesion from degenerating.

When quantifying changes seen in OCT images, our first attempt to segment OCT B-scans using the software included in the HEYEX 2 OCT equipment failed (Supplemental Figure 1). This prompted us to develop and train deep learning (DL) algorithms to perform segmentation on porcine retinal images. Our DL algorithm pipeline involved the use of 3 separate U-Net models (https://arxiv.org/abs/1505.04597) to segment the 3 distinct categories of porcine retina in our study: U-Net #1 was trained to segment 6 layers in healthy retinas (nerve fiber layer [NFL]; ganglion cell layer/inner plexiform layer [GCL/IPL], inner nuclear layer [INL], ONL, EZ, interdigitation zone/RPE); U-Net #2 was trained to segment retinal layers in addition to the damaged zone (DZ) — retinal layers with no clear demarcation and boundaries because of the laser injury (Supplemental Figure 2); U-Net #3, a binary DL model, was trained to discriminate transplanted regions from the remainder of the retina (Supplemental Figure 3).

Each algorithm was trained, validated, and tested with distinct datasets of B-scan images: U-Net #1 used a total of 478 images (267 training images, 67 validation images, and 144 test images), U-Net #2 used 666 images (378 training images, 90 validation images, and 198 test images), and U-Net #3 used 317 images (179 training images, 40 validation images, and 98 test images). Each U-Net algorithm’s parameters were optimized based on a quantitative and qualitative comparison of numerous architectures. After training, algorithm performance was first qualitatively evaluated by comparing it to the ground truth. In the qualitative assessment of the DL algorithm’s performance, U-Net #2 was found to correctly segment retinal layers and the DZ for most images (Supplemental Figure 2, A–C). However, errors were occasionally observed in images with retinal vessel shadows, which tended to obscure inner retinal layer boundaries (Supplemental Figure 2, D–F). Furthermore, we rarely also observed retinal displacement, with layers appearing out of their typical order or axially displaced, likely due to the presence of hyporeflective choroidal blood vessels (Supplemental Figure 2, G–I). These errors were manually corrected before proceeding with quantitative analysis of retinal layers.

U-Net #3 showed a high level of accuracy in most cases (Supplemental Figure 3, A–C). However, 2 types of errors were noted in the qualitative assessment: transplant fragmentation and errors due to hyper-reflective debris. Fragmentation errors were occasionally noted where the continuity of the transplant seemed slightly irregular (Supplemental Figure 3, D–F). To fix these errors, graders used the HEYEX 2 software to reference fundus images when manually determining boundaries on OCT B-scans. Hyper-reflective debris, a consequence of laser injury, often led to an overestimation of the transplant area in the model predictions and was manually corrected (Supplemental Figure 3, G–I).

**Figure 3:**
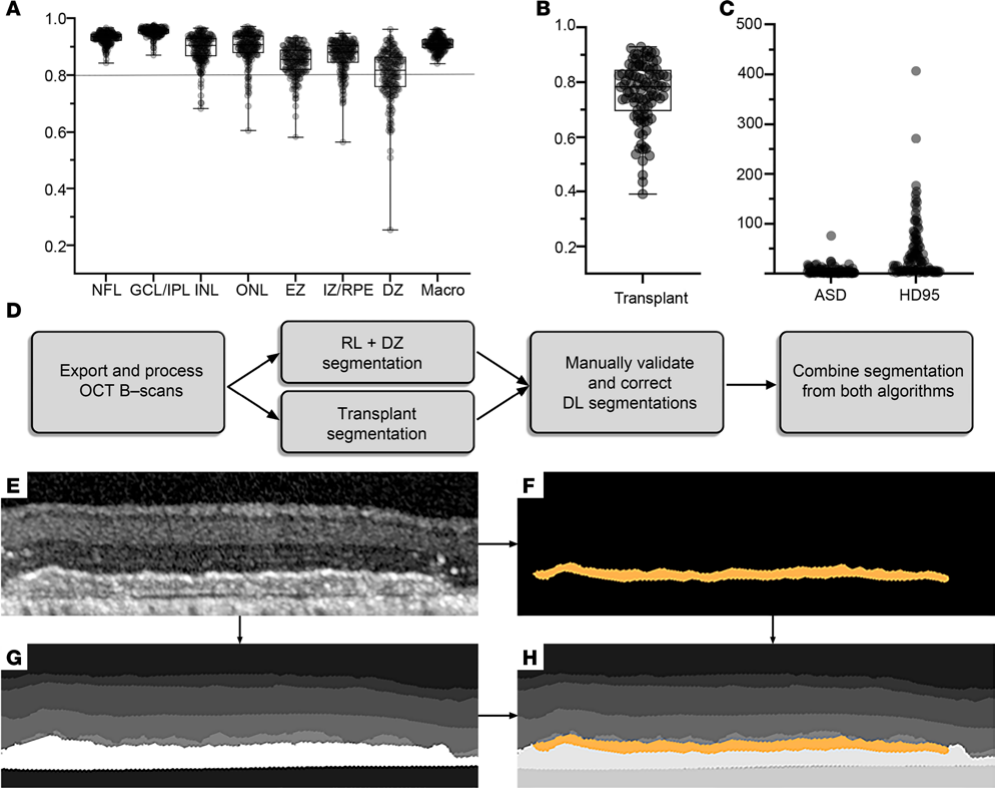
Development of DL networks for segmentation of OCT images. (**A** and **B**) Dice coefficients representing spatial overlap between predicted and ground truth segmentation for U-Net #2 (**A**) and U-Net #3 (**B**). (**C**) Individual values of ASD and HD95 for segmentation of PLGA-iRPE and/or PLGA-only transplants by U-Net #3. (**D**) Process of combining U-Net #2 and U-Net #3 to segment retinal layers, DZ, and transplant(s) in a single OCT B-scan. (**E**–**H**) Representative OCT images of an unsegmented image containing a PLGA-iRPE transplant, retinal layers, and DZ (**E**); PLGA-iRPE segmentation generated by U-Net #3 (**F**); correctly segmented image of retinal layer and DZ segmentation generated by U-Net #2 (**G**); and the resulting overlay of all the corrected segmentation images combining data from both U-Nets (**H**). Box and whiskers represent min and max, 25th and 75th percentile, median, and single values.

Algorithm performance was quantitatively evaluated using 3 independent metrics: (i) dice coefficient, which assesses segmentation accuracy by considering total area and area of overlap between ground truth and U-Net predictions (20); (ii) average surface distance (ASD), which represents the average discrepancy between the boundaries of the ground truth and U-Net segmentation; and (iii) 95th percentile Hausdorff Distance (HD95), which indicates the 95th percentile of discrepancies between the predicted and ground truth boundaries (21). Interrater variability analysis, assessing consistency among human raters, yielded a macro dice coefficient of 0.94 for images segmented by U-Net #1 and 0.87 for those by U-Net #2, while the dice coefficient for transplant segmentation in U-Net #3 images was 0.81. For U-Net #1 and U-Net #2, the mean of the dice coefficient was over 0.8 for each of the retinal layers, suggesting robust and accurate segmentation (Figure 3A and Supplemental Table 1). The overall macro dice coefficients for U-Net #1 and U-Net #2 were 0.95 and 0.90, respectively. These results are comparable to or exceed the interrater dice coefficient, suggesting these U-Nets show consistency comparable to human raters. Similarly, U-Net #3 segmented the transplant with an average dice coefficient of over 0.75, marginally lower than the interrater dice coefficient, indicating similar or slightly less consistent performance (Figure 3B and Supplemental Table 2). ASD and HD95 were primarily utilized in addition to dice coefficient in circumstances where the algorithm was used to segment structures that did not span across the entirety of the OCT B scan volume (e.g., PLGA-only and PLGA-iRPE). Further evaluation of the metrics suggested accurate and reliable segmentation of the transplant through U-Net #3, as the median ASD was 2.77 pixels (0.030 mm), and the mean HD95 was 44.92 pixels (0.49 mm) (Figure 3C and Supplemental Table 2). Moreover, the segmentation of the transplant by U-Net #3 was successfully overlaid onto the segmentation by U-Net #2, allowing an accurate segmentation and subsequent quantification of retinas containing all retinal layers, DZ, and transplants (Figure 3, D–H). Overall, our analysis suggests that our U-Net algorithms were robustly trained, allowing us to perform quantifiable assessment of different retinal layers more efficiently in baseline, lasered, and transplanted regions.

**Figure 4:**
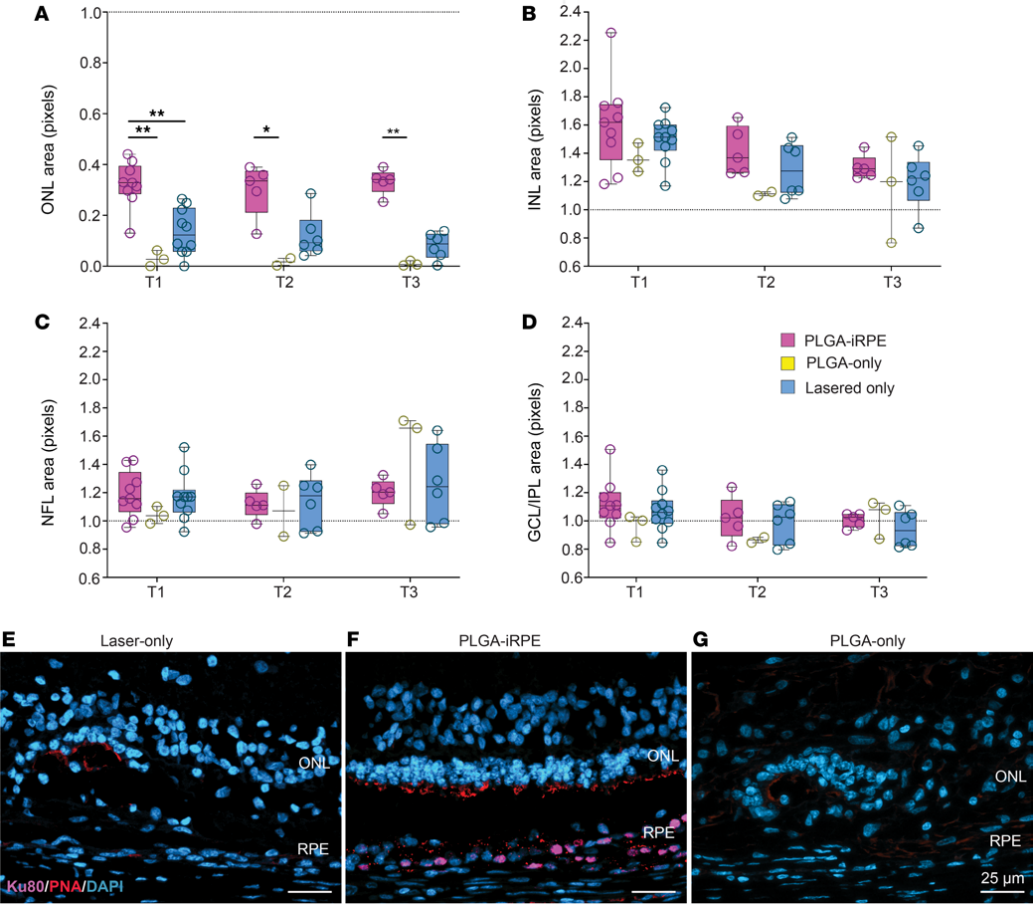
DL algorithm-based quantification of OCT images. (**A**–**D**) Area of the ONL (**A**), INL (**B**), NFL (**C**), and GCL/IPL (**D**) in PLGA-iRPE (pink), PLGA-only (yellow), and laser only (blue) at 3 time points: T1, T2, T3. Data at T1, T2, and T3 were normalized to baseline values. *P* values are reported as *P < 0.05; **P < 0.01. (**E**–**G**) Cross-sectional views of swine retina from laser-treated (**E**), laser-treated and transplanted with PLGA-iRPE (**F**), and laser-treated and transplanted with PLGA-only (**G**), performed at time point T3, stained for DAPI (blue) to detect nuclei and PNA (red) to detect cone photoreceptor outer segments and immune-stained for Ku80 (pink) to detect human cells and PMEL17 to detect RPE melanosomes. The Friedman test was used to assess repeated measures data, and the Dunn test was used to compare groups at each time point. Scale bars: 25 μm. PNA, peanut agglutinin.

Quantitative analysis of segmented OCT images revealed that at all 3 time points, ONL thickness was significantly different between PLGA-iRPE and PLGA-only. PLGA-iRPE–transplanted retinal regions showed approximately 3-fold higher ONL thickness as compared with the lasered area (*P* < 0.01 for T1) and approximately 16-fold higher as compared with the PLGA-only (*P* < 0.01 for T1 and T3, and *P* < 0.05 for T2) retinal region (Figure 4A). These data suggest that the transplanted human iRPE monolayer protects photoreceptors in the lasered area from degenerating. In contrast, the PLGA-only transplant does not protect the photoreceptors. There was no statistically significant difference in the thickness of NFL, GCL/IPL, or INL layers when comparing lasered, PLGA-only transplant, and PLGA-iRPE transplant regions (Figure 4, B–D). The ability of transplanted human iRPE monolayer to protect photoreceptors was further verified by histological analysis, at the terminal time point (T3) (Figure 4, E–G). Ku80 and PMEL17 immunostaining verified the presence of human RPE cells only in PLGA-iRPE–transplanted retina (Figure 4F). DAPI staining revealed preservation of photoreceptor ONL, whereas PNA staining showed photoreceptor outer segments only over the area of transplanted PLGA-iRPE and not over PLGA-only transplants (compare Figure 4, F and G). These data underscore the dependency of photoreceptors on healthy RPE for survival. Overall, our quantification data support the qualitative OCT assessment, indicating that human iRPE cells as a PLGA-iRPE transplant preserve pig photoreceptors.

### PLGA-iRPE rescues cone photoreceptor density as imaged by AO.

**Figure 5:**
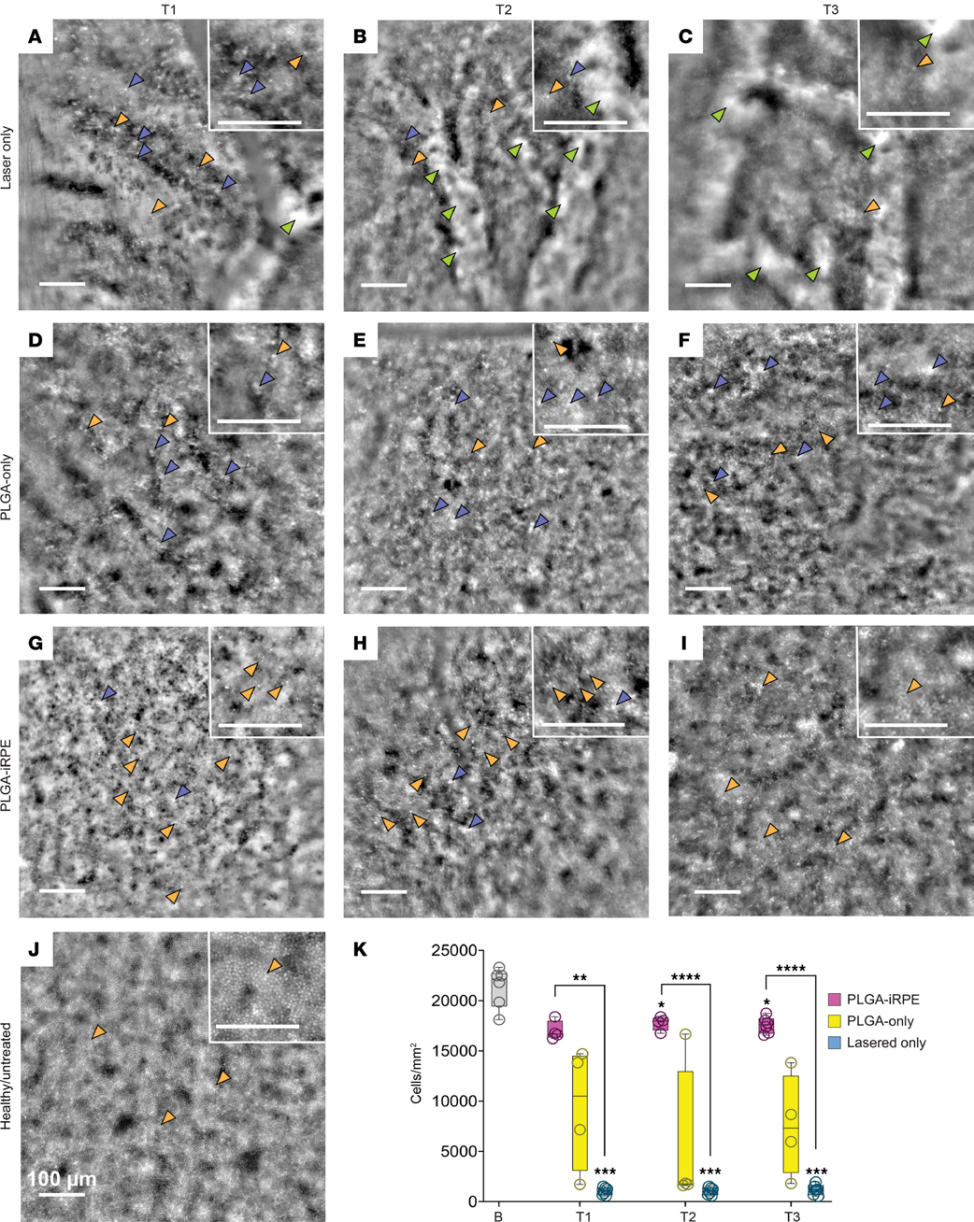
AO-based evaluation of cone photoreceptor density. (**A**–**I**) AO images of the retina after laser injury (**A**–**C**), after PLGA-only transplant (**D**–**F**), and after PLGA-iRPE transplant (**G**–**I**) at time points T1 (**A**, **D**, and **G**), T2 (**B**, **E**, and **H**), and T3 (**C**, **F**, and **I**). (**J**) AO image of baseline retina. For each panel (**A**–**J**) a small area is magnified for better visualization of choroidal vessels (green arrowheads), cone photoreceptors (orange arrowheads), and cell debris (blue arrowheads). (**K**) The cone photoreceptor cell density analysis at baseline (gray) and in regions of laser injury (blue), PLGA-iRPE (pink), and PLGA-only (yellow) transplants. Box and whiskers represent min and max, 25th and 75th percentile, and median and single values. Data were analyzed with a mixed-effect model (restricted maximum likelihood, REML) and Tukey’s multiple comparisons. A total of 5 regions of interest in 3 images for each eye were analyzed. Number of eyes used for the analysis (*n*) = 5 T1, 5 T2, 4 T3 for PLGA-iRPE; 4 T1, 5 T2, 4 T3 for PLGA-only; 7 T1, 7 T2, 8 T3 for laser. *P* values are reported as *P < 0.05; **P < 0.01; ***P < 0.005; ****P < 0.0001.

To corroborate OCT findings at a single-photoreceptor level, we utilized AO analysis, enabling longitudinal single-cell resolution imaging of cone photoreceptors (Figure 5). Representative images at baseline and time points T1, T2, and T3 are shown in Figure 5, A–I. The bright circular dots, each corresponding to a single cone photoreceptor, showed a statistically significant reduction in number after the laser injury (orange arrowheads, Figure 5, A–C). This suggests cone photoreceptor cell death, as shown previously by correlative analysis of AO and histological data (19, 22). Choroidal vessels are visible as a bright signal (green arrowheads, Figure 5, A–C), suggesting a loss of RPE monolayer. Analysis of retina over PLGA-only transplants revealed a higher prevalence of retinal hyper-reflective foci described previously as cell debris (19) and a reduced number of bright circular dots that correspond to cone photoreceptors (orange arrowheads, Figure 5, D–F). In contrast, analysis of retina over PLGA-iRPE transplants showed negligible hyper-reflective foci and a statistically significant increase in number of cone photoreceptors (orange arrowheads, Figure 5, G–I), underscoring the ability of PLGA-iRPE to preserve cone photoreceptors. In comparison with these treatments, at baseline, cone photoreceptors were visible as bright circular dots distributed in an orderly manner (Figure 5J, orange arrowheads). To quantify changes in cone photoreceptor cell density, we counted the number of cone photoreceptors using rtx1 imaging equipment software in images taken from healthy and laser-treated retina, as well as retina over PLGA-only and PLGA-iRPE transplants (Supplemental Figure 4, Figure 5K, and Supplemental Figure 4, G and H, show background reflectance caused by PLGA-only transplants). Quantification of the cone photoreceptor cell density validated our visual observations. At all 3 time points (T1, T2, and T3) the cone photoreceptor cell density over PLGA-iRPE was approximately 17-fold higher (*P* < 0.01 at T1, and *P* < 0.0001 at T2 and T3; Figure 5K) as compared with lasered area, with an average of 17,000 photoreceptors/mm^2^ over PLGA-iRPE and 1,000/mm^2^ in the lasered area (Figure 5K). In contrast, the density of photoreceptors over the PLGA-only transplant was variable, with an average of 7,000 cells/mm^2^ (Figure 5K). Averages and standard deviations of cone photoreceptor density are reported in Supplemental Table 3. Overall, our longitudinal single-cell-based analysis of cone photoreceptors validated OCT data that the human iRPE-patch performs better as compared with PLGA-only transplants in protecting pig photoreceptors from degeneration after laser ablation of RPE.

### PLGA-iRPE promotes choriocapillaris reperfusion.

**Figure 6:**
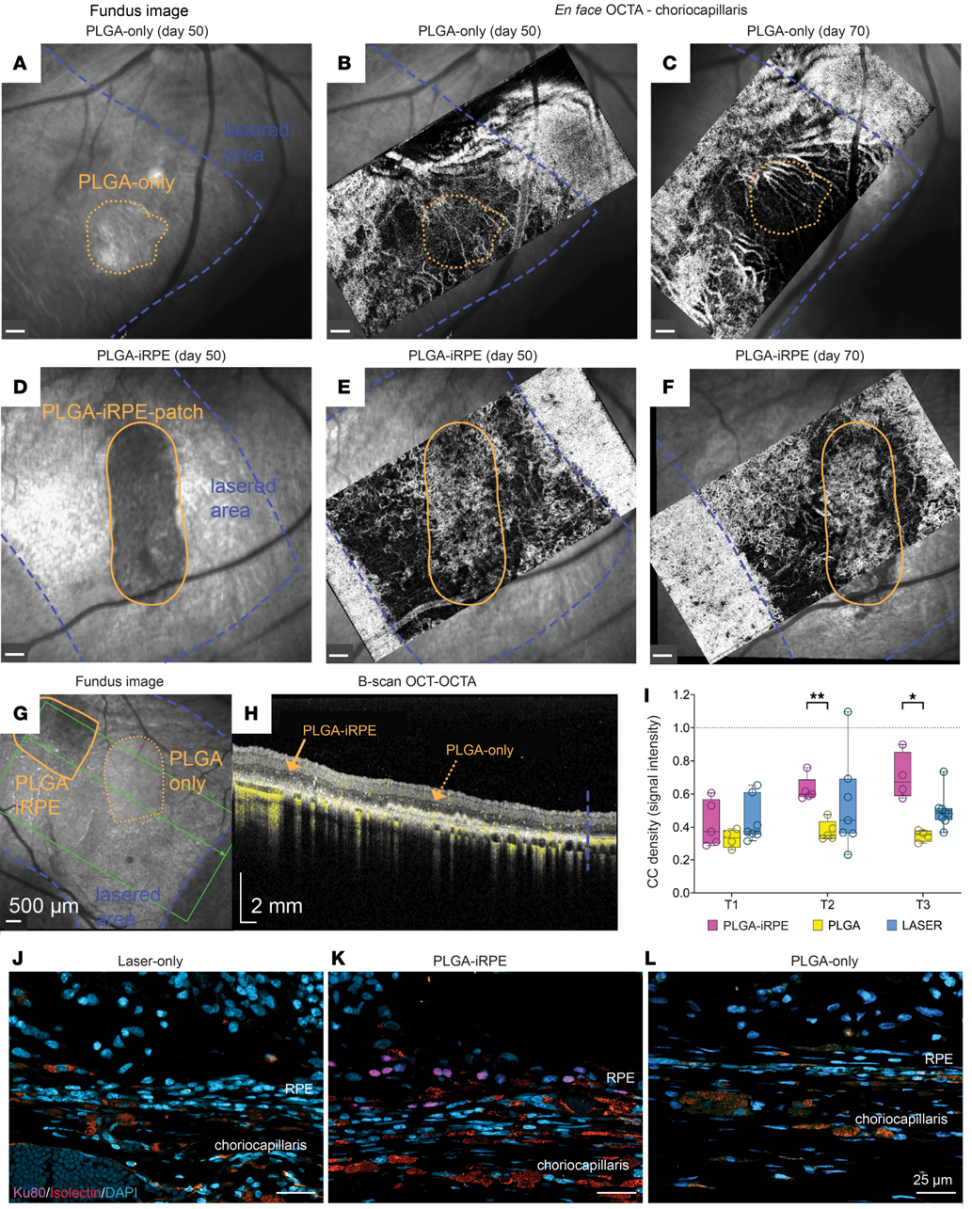
OCT-A–based evaluation of choriocapillaris perfusion. (**A**–**F**) PLGA-only–transplanted (**A**–**C**) and PLGA-iRPE–transplanted (**D**–**F**) laser-injured retinas showing the location of the transplant in fundus infrared images (**A** and **D**) and en face OCT-A images of choriocapillaris signal at 50 days (**B** and **E**) and 70 days (**C** and **F**). (**G** and **H**) Fundus infrared image (**G**) of PLGA-only– and PLGA-iRPE–transplanted retina. The lasered region in **A**–**G** is marked with a blue dotted line, PLGA-only with an orange dotted line, and PLGA-iRPE with a solid orange line. The green line in **G** shows the location of the OCT-A B-scan in **H**. The corresponding B-scan (**H**) shows the choriocapillaris signal marked in yellow. (**I**) Quantification of choriocapillaris signal segmented from OCT-A B-scans under the laser-injured and PLGA-only– and PLGA-iRPE–transplanted retinas. Box and whiskers represent min and max, 25th and 75th percentile, median, and single values. Data were analyzed with a mixed-effect model (REML) and Bonferroni’s multiple comparisons, and 3 images for each eye were analyzed. Number of eyes used for the analysis (**I**) (*n*) = 5 T1, 5 T2, and 4 T3 for PLGA-RPE; 4 (T1), 5 (T2), and 4 T3 for PLGA; 7 T1, 7 T2, and 8 T3 for laser. T1 = days 12–28, T2 = days 29–45, T3 = days 46–80. *P* values are reported as *P < 0.05; **P < 0.01. (**J**–**L**) Cross-sectional views of swine retina from laser-treated (**J**), laser-treated and transplanted with PLGA-iRPE (**K**), and laser-treated and transplanted with PLGA-only (**L**), stained for DAPI (blue) to detect nuclei and isolectin (red) to detect choriocapillaris, and immune-stained for Ku80 (pink) to detect human cells. Scale bars: 25 μm.

It is thought that degenerated choriocapillaris in the late stages of dry AMD may limit the survival of transplanted RPE because of a lack of nutrient supply (18). To investigate the effect of our PLGA-iRPE on choriocapillaris health, we used OCT-A analysis. Representative en face OCT-A images of the segmented choriocapillaris beneath the PLGA-iRPE and the PLGA-only transplants are shown in Figure 6, A–F. Lasered and transplant retinal regions were located using fundus imaging (Figure 6, A and D, dotted and solid orange lines). Comparative analysis of PLGA-only and PLGA-iRPE transplants showed missing choriocapillaris perfusion beneath the transplant at time point T1, as verified by a lack of bright signal (Supplemental Figure 5). However, starting at day 50, statistically higher choriocapillaris regeneration was noted under the PLGA-iRPE as compared with PLGA-only transplants, which showed negligible choriocapillaris reperfusion (dotted and solid orange lines; Figure 6, B and E). Choriocapillaris reperfusion underneath the PLGA-iRPE was stable through to the last evaluation time point (day 70; solid orange lines; Figure 6, C and F). To quantify these qualitative findings, we used OCT-A scans (Figure 6, G and H, and Supplemental Figure 6). Quantification verified that as compared with PLGA-only transplants the choriocapillaris signal under the PLGA-iRPE transplant increased over time and was higher at T2 and T3 (Figure 6I). Raw data of choriocapillaris pixel values expressed as mean and standard deviation are reported in Supplemental Table 4. To determine if OCT-A findings could be confirmed histologically, retina sections were stained with isolectin-IB4 to label capillaries and blood vessels and immunostained for Ku80 to identify iRPE-transplanted regions (Figure 6, J–L). Histological findings support the OCT-A data, revealing high density of choriocapillaris only underneath iRPE-transplanted regions and not in laser-treated or PLGA-only–transplanted regions. Overall, OCT-A and histology data verified that choriocapillaris reperfused specifically underneath the human PLGA-iRPE and not underneath the PLGA-only region.

## Discussion

We developed a surgical procedure to achieve immediate sequential delivery of 2 transplants (PLGA-only and PLGA-iRPE) in adjacent subretinal locations, thereby allowing direct comparison of the retinal regions surrounding each implant. This work provided proof of concept, showing that a double transplant dose (2 times 8 mm^2^) can be successfully delivered. A critical component of this surgery was the use of a custom surgical clamp that allowed maintenance of eye pressure while 2 patch delivery cannulas were alternated in the eye. Postoperative retinal and choroidal status were monitored using clinically relevant modalities including OCT, AO, and OCT-A. Manual and DL-based segmentation allowed quantification of changes in retinal layers and choriocapillaris, providing strong and direct proof of PLGA-iRPE efficacy, including its ability to slow down laser injury–induced retinal degeneration and to regenerate laser-induced degeneration of choriocapillaris. The retina over the 2 transplants reattached similar to the reattachment observed over a single transplant (10). Furthermore, the retinotomy site used for the delivery of 2 transplants healed well. Together, these 2 observations support surgical feasibility of 2 transplants in a single eye without additional damage to the retina.

Two key surgical steps ensured the success of this dual-transplant surgery: (i) The PLGA-only and PLGA-iRPE transplants were loaded in separate cannulas and stored in a surgical excipient inside the sterile field of the surgical suite to ensure transplant sterility and readiness for delivery; (ii) the sclerotomy was kept sealed, preventing hypotony. This was achieved by using our customized clamp that grips into the sclera, keeping the sclerotomy watertight, while allowing the simultaneous insertion of the second cannula. The clamp was designed with a slight opening to allow cannula insertion, preventing compression of transplants and RPE cells during delivery. In a small number of cases, the clamp was unable to close the wound effectively for the required time needed to deliver both implants. Based on our experience, implementing a strategy to optimize the placement of the scleral clamp, ensuring it grasps the thicker segment of the sclera, can significantly enhance surgical outcomes.

Delivery of 2 transplants adjacent to each other in the same eye provides several benefits. (i) It allows direct comparison of the test transplant with the sham control in the same eye in a preclinical animal model, reducing the eye-to-eye variability seen in data when comparing PLGA-only with test transplant delivered in different eyes or animals. (ii) It allowed us to monitor relative local immune response against the scaffold versus the xeno-cells. (iii) It also allowed us to directly compare potential efficacy between the PLGA-only and PLGA-iRPE transplants, providing supporting mechanistic evidence for our cell therapy. Multimodal image analyses of the retina over the area of 2 transplants clearly show that PLGA-iRPE as part of a scaffold is more efficacious as compared with PLGA only, supporting the biological observation of the ability of RPE cells to support photoreceptor and choriocapillaris health (19, 23). (iv) In patients, this will allow us to deliver a double dose of the transplant (2 times 8 mm^2^), covering the majority of human macula of 19.5 mm^2^ (11). (v) This approach opens the possibility of treating large zones of geographic atrophy, potentially of different sizes and shapes, with multiple transplants targeting contiguous or noncontiguous areas (8, 11). (vi) In preclinical animal models, this allows delivery of different types of transplants (RPE derived from different stem cell sources, RPE cultured on different types of scaffolds, photoreceptor subtypes, RPE/photoreceptor combined transplants) in different locations of the retina, thus opening the possibility of treating different areas of the retina with different types of cell therapies.

Immune suppression was achieved by a combined cyclosporine, mycophenolate mofetil, and steroid-based regimen. High blood levels of cyclosporine (>700 mg/mL) have been correlated with central nervous system, pulmonary, and renal toxicity in pigs (24). Hence, cyclosporine levels were tightly regulated by weekly analysis of animals’ blood samples. Despite an identical body weight–based dosing of cyclosporine, its levels ranged from 140 ng/mL to 1,952 ng/mL, likely because of individual differences in metabolism. Although the biological basis for variable cyclosporine metabolism is not clear, its levels are directly associated with host immune response against the transplant. When cyclosporine levels dropped below 250 ng/mL, animals presented an immune response, seen on OCT as a hyporeflective signal underneath the PLGA-iRPE, prompting us to adjust its levels, which often resulted in a reduced immune response against the transplant. Our data suggest close monitoring of cyclosporine levels is critical when testing human cell xeno-transplants in pigs.

The software provided by Heidelberg Engineering, HEYEX 2, was not designed to work on porcine retinas. This problem was further compounded by the laser injury and transplant delivery, as both treatments significantly altered the retinal anatomy. This prompted us to develop our own algorithm so that we could directly compare the potential efficacy of the 2 transplants. The rationale for the choice of 3 separate DL models is based on previous work by Kugelman et al. (25), which suggests that a specific DL model is most effective when trained to segment a specific disease or type of retinal degeneration. Based on this literature evidence, a third DL model (U-Net #3) was developed and overlaid onto the results from the second model (U-Net #2), allowing for segmentation of the transplant while maintaining retinal layer and DZ segmentation. We anticipate that such models, trained specifically for the intended animal, will further improve the rigor of preclinical testing in large animals. Due to severe retina damage in the lasered area, the EZ layer was indiscernible from the transplant and/or surrounding debris and therefore nonsegmentable. Consequently, changes in the EZ region within lasered or transplanted retinas could not be quantified. This necessitated a new retina classification (DZ) during OCT image segmentation.

Analysis of OCT data was further corroborated by AO analysis that revealed a higher number of individual cone photoreceptors over the PLGA-iRPE as compared with the PLGA-only transplant, though often this recovery was noncontinuous. This noncontinuous recovery, combined with a limitation of AO analysis, precluded correct visualization of regularity and number of neighbors for cases where the photoreceptor cell density was too low; this effect was more pronounced for the laser-injured and PLGA-only–transplanted retina. This issue presented itself when using the Voronoi method of cell order analysis; as cell density diminished, the resulting tessellating polygons became irregularly shaped and failed to accurately represent the morphology of the cells. In addition, we believe, PLGA-only transplant increases background reflectance in AOimaging, hence increasing the background signal that is measured as highly variable signal on AO. It is also worth noting despite potentially discrepant results between AO and OCT analysis, there was no significant difference between PLGA-only and laser at any time point in both AO and OCT analyses. These limitations may have resulted in discrepancy when comparing OCT and AO results of lasered and PLGA-only–transplanted retinas. Furthermore, we speculate that PLGA-only transplant interferes with nutrient supply to photoreceptors, exacerbating their degeneration. This idea is supported by our previous observation where transplantation of a PLGA-only transplant under a healthy retina resulted in degeneration of photoreceptors (10).

It has long been suggested that RPE cells maintain the health of choriocapillaris and that in advanced AMD stages RPE cell death triggers choriocapillaris atrophy (18). It is, however, not clear if RPE cell death–induced choriocapillaris atrophy can be reversed using an RPE transplant or if RPE cells succumb because of lack of nutrient support in areas of choriocapillaris atrophy. This study provides the first direct evidence to our knowledge that a PLGA-iRPE and not PLGA-only scaffold is able to regenerate atrophied choriocapillaris-like perfusion in the lasered region. Underlying choroidal vessels are the likely source of these regenerated choriocapillaris; however, our work does not provide any direct evidence to support this hypothesis.

One drawback of our laser-injury model is the gradual ingrowth of native RPE cells from the periphery of the laser lesion. However, this native RPE cell proliferation process is slow, often taking several weeks to become evident (19). To circumvent any confounding effects from native RPE ingrowth, we strategically transplant the PLGA-iRPE patch into the central portion of the lasered region, where we start observing its capacity to halt photoreceptor degeneration within 2 weeks posttransplantation. Our surgical procedure was limited by the thin scleral anatomy in pigs, which is prone to wound leakage during surgery, but leakage can be overcome with a wound closure clamp. In cases where the wound could not be kept watertight, failed delivery of the 2 transplants resulted. Depending on the size and location of retinal detachment, another limitation is the occasional inability to control the location of the 2 transplants. In the future, we plan to extend this work and compare the relative efficacy of different RPE subpopulations (macular cells versus peripheral cells) and RPE-only transplants with dual RPE/photoreceptor transplants (26).

## Methods

### Sex as a biological variable

Only castrated male minipigs (Yucatan, 35–45 kg) from Premier BioSource and Sinclair Research with homogeneous fundus pigmentation were included in the study. Females were not included because we do not have male-female cohousing, breeding facilities, and protocols. It is unknown whether the findings are relevant for any specific sex.

### Animal housing

All animals in this study were housed and treated according to the NIH and Association for Research in Vision and Ophthalmology guidelines for the humane treatment of animals in visual research. Animals were singly housed in large indoor enclosures (pens, approximately 6 feet by 10 feet), were housed in climate control rooms with wood shavings on the floor, had food administered 3 times/d, and had water offered ad libitum.

### Study design

PLGA-iRPE patch was developed and validated as reported in our previous work (10). PLGA-iRPE and PLGA-only transplants were delivered 2 days after the laser-induced ablation of pig RPE, performed using a 1% duty cycle (DC) 532 nm micropulse laser (1,500–1,800 mW), as described in Barone et al. 2023 (19). Laser ablation and transplantation were performed within the porcine visual streak, a region containing a higher density of cone photoreceptors as compared with the peripheral retina, which is comparable to the human macula (27).

All pigs received a presurgery baseline examination, which included clinically relevant imaging modalities, including fundus infrared (IR), OCT, AO, OCT-A. The same set of examinations was repeated during each follow-up. Follow-ups were repeated at least once in each of the following time point ranges after transplantation: T1 (12–28 days), T2 (29–45 days), and T3 (46–80 days).

For imaging, laser injury, or vitreoretinal surgery, pigs were premedicated using glycopyrrolate (0.01 mg/kg, i.m., American Reaent Inc); after 20–30 minutes, anesthesia was induced by i.m. administration of telazol (5 mg/kg, Zoetis) mixed with butorphanol (0.2 mg/kg, Torbugesic, Zoetis) and dexmedetomidine (0.035 mg/kg, Dexmedesed, Dechra). Animals were then moved to the procedure room and intubated, and an intravenous catheter was placed. Inhalant anesthesia was maintained with isoflurane or sevoflurane while animals were mechanically ventilated under a pressure/volume-controlled setting. Pigs were positioned in dorsal decubitus in custom cradles. Water and air-warming blankets were used to maintain the body temperature. Blood pressure, heart rate, blood oxygenation, CO_2_, and temperature were monitored continuously. Sodium chloride (0.9% sodium chloride injection USP, Hospira) or lactated ringer (Lactated Ringers injection USP, ICU Medical) solutions were administered i.v. throughout the procedure. Pupils were dilated with tropicamide 1% (tropicamide ophthalmic solution 1% USP, Akorn or Sandoz) and phenylephrine 10% (phenylephrine hydrochloride ophthalmic drops 10% USP, Paragon Biotech). During image collection and laser injury, rocuronium (2–3 mg/kg, i.v., rocuronium bromide injection 10 mg/mL USP, XGen) was administered as needed for relaxation of the extraocular muscles. Upon completion of imaging, an ophthalmic ointment (Neomycin and Polymyxin B sulfates ophthalmic ointment USP, Bausch & Lomb) was placed on the corneal surface. Ketoprofen (3 mg/kg, i.m., Ketofen 100 mg/mL, Zoetis) was given to reduce muscle pain related to paralytic administration. Fluorescein and indocyanine green were administered i.v.

### Anesthesia protocol for enucleation and euthanasia

Animals were anesthetized using the protocol outlined above, intubated, and maintained on a pressure-controlled ventilator. Following enucleation, pigs were euthanized immediately by administering B-euthanasia i.v. 1 mL/10 lbs. BW (Euthanasia solution, VetOne). Animals’ heart rate, blood pressure, and respiration were monitored to ensure euthanasia.

### Immune suppression regimen and cyclosporine monitoring

The protocol was as follows. Cyclosporine 12–20 mg/kg/d orally (PO) started 3 days before surgery and continued until euthanasia, weekly alternating between 12 and 20 mg/kg/d. Mycophenolate mofetil 500 mg/d PO started 3 days before surgery, continuing until euthanasia. Prednisone 5 mg/kg/d PO started 3 days before surgery, continuing until euthanasia. Sulfamethoxazole trimethoprim 30 mg/kg/d PO started 2 days before surgery, continuing until euthanasia. Kineret 100 mg/d s.c. started 3 days before surgery, for 11 days total. Apoquel (weight-based chart) PO started the day before surgery, continuing until euthanasia. Methylprednisolone/Solu-Medrol 500 mg i.m. single dose was given on the surgery morning. Cyclosporine levels were measured by Antech Diagnostics. For these measurements, 3 mL of animal blood was shipped.

### Laser injury

The laser injury technique used in this study was previously reported by Sharma et al. and Barone et al. (10, 19). A 532 nm micropulse green laser (Iridex) equipped with TxCell technology was used, as this specific wavelength is efficiently absorbed by the RPE melanin. As opposed to continuous-wave lasers, in micropulse mode the exposure time is divided into pulses called DCs with a short ON time and a long OFF time; this characteristic allows tissue to cool down between pulses, thus limiting the area of tissue damage to the RPE and vicinity. In addition, TxCell technology allows confluent lesions to be made in the treated area. Laser power was adjusted to reach the minimum visible threshold (outside the planned surgery area) to compensate for the natural changes in the pigmentation of the pig eyes (16). A spot size of 200 μm, 330 ms of exposure time, and a DC of 1% (0.1 ms ON/9.9 ms OFF) were used with laser power of 1,500–1,800 mW. Confluent threshold (visible) lesions were performed to cover an area of approximately 50 mm^2^ along the retinal central streak. The threshold was tested as a whitening of the retina corresponding to the pulse location.

### Surgery and dual scaffold transplantation protocol

A standard 3-port 25G pars plana vitrectomy was performed with posterior vitreous detachment. Using a 25G/38G cannula, a localized retinal detachment was induced using Healon BSS at the target transplantation site, and a scissors retinotomy was subsequently completed to access the subretinal space. Through a 2.4 mm sclerotomy, a custom-made cannula loaded with either the PLGA-only or PLGA-iRPE transplant was delivered to the targeted subretinal space. After placement of the first transplant, the cannula was withdrawn, and the eye was closed using the custom scleral clamp. Watertight closure was verified while the cannula containing the second scaffold was handed to the surgeon. The clamp was released immediately prior to cannula introduction and was replaced as soon as the cannula was withdrawn following the second transplant placement. Transplant placement was verified with intraoperative transpupillary OCT after placement of both constructs. The delivery of the transplants was followed by fluid-air exchange to flatten the retina; air was exchanged with long-term gas (15% C3F8) to maintain the retinotomy closure until healing. The retinotomy edges were not lasered. The sclerotomy and ports were sutured with 8-0 nylon sutures (Microsurgical).

### Image acquisition

#### OCT.

All OCT volumes were obtained using the Spectralis spectral-domain–OCT (SD-OCT) instrument (Heidelberg Engineering). At least 2 volumes were taken during each evaluation session; OCT volumes were centered on the transplant(s), and the raster lines were aligned either parallel (longitudinal) or perpendicular (transverse) to the visual streak. Each OCT volume contained a corresponding fundus IR image where OCT volume boundaries and B-scan location were marked. Eye tracking technology allowed follow-up scans to be recorded in the same location. OCT volumes were taken in high-resolution mode to improve signal-to-noise ratio. Each B-scan was averaged between 15 and 18 times. Furthermore, volumes contained between 55 and 217 raster scans. B-scans measured 1,536 pixels wide and 496 pixels deep with transverse and axial resolutions of 10.8 μm/pixel and 3.87 μm/pixel, respectively, which correspond to a physical dimension of approximately 16.6 mm × 1.9 mm per B-scan. Selected B-scans were exported individually in TIF format (no compression) with the corresponding fundus image using the HEYEX 2 software (Heidelberg Engineering).

#### OCT-A.

OCT-A volumes were centered on transplanted scaffolds and were obtained with the Spectralis SD-OCT instrument. Volume scans were performed between 512 and 768 scans and were 496 pixels deep with mean transverse and axial resolutions of 12.16 μm/pixel and 3.87 μm/pixel, respectively. On average, these properties correspond to a physical dimension of approximately 9.2 mm × 1.9 mm per scan. Selected scans were exported individually in TIF format (no compression) using the HEYEX 2 software. Exported images are composed of a corresponding fundus image and all OCT/OCT-A scans at the identical location.

#### AO.

The rtx1 equipment (Imagine Eyes) was used for AO-flood illumination ophthalmoscopy. The equipment was manually adjusted by tilting the cradle on the vertical axis and rotating the rtx1 machine in the horizontal plane to capture specific regions of interest (ROIs) within the retina. At least 2–3 montages were collected for each ROI, and 5–10 images were collected for each montage.

### AO image analysis

Montages of AO images were generated with i2K retina software and were subsequently superimposed with fundus IR and FA images. IR fundus images better outlined laser or transplant locations, while FA images allowed for improved alignment of the AO images on the retinal and choroidal vasculature. In baseline images, three 80 pixel × 80 pixel squares were randomly sampled for quantification from a single montage. In laser, PLGA-only, and PLGA-iRPE images, five 80 pixel × 80 pixel squares were sampled to better represent the variability in cone photoreceptor density in heterogeneous appearing regions. The analysis included squares exclusively from regions deemed to have acceptable focus. Parameters of density and regularity were averaged for each group of 3 or 9 squares. Counting of cone photoreceptors was carried out using the AOdetect software (AOdetect Mosaic, Imagine Eyes). Three graders verified its accuracy.

### Image organization for the training of DL algorithms for OCT segmentation

The baseline dataset was composed of 28 longitudinal volumes (28 eyes; 14 animals), and the laser dataset was composed of 31 longitudinal volumes (20 eyes; 13 animals); each volume was composed of 18 B-scans. For volumes in the healthy nonlasered dataset, B-scans were evenly split throughout the middle of the volume. The scans at the outer limits of the volume were not included because of commonly observed B-scan truncation in these areas. In the laser dataset, B-scans were evenly spread between the first and last B-scans with laser-induced atrophy. The transplant dataset was composed of 32 volumes (15 eyes; 13 animals), with 10 B-scans in each volume). The volume was split evenly between transverse and longitudinal OCT scans (5 transverse and 5 longitudinal in each volume), as there was varied alignment of the scaffold with the visual streak. B-scans were spread evenly within the boundaries of the transplant. In OCT B-scans, the edges of transplants were difficult to discern from hyper-reflective debris, likely because: 1) the sync between fundus image to OCT is not perfect; and 2) edges tend to integrate faster than the rest of the transplant. Hence, the boundaries of the transplant were not included in the dataset.

### DL training, validation, and testing data sets

All datasets were segmented using LabelMe, a Python-based annotation tool (https://github.com/labelmeai/labelme) by one trained operator. For each dataset, segmentation was repeated by another operator on 75 randomly selected images to analyze interrater reliability. To assess the performance of each DL model, datasets were separated randomly into training, validation, and test subsets. For U-Net #1, volumes were split by animal, such that volumes or scans from the same animal could not be present in more than one subset. For U-Net #2 and U-Net #3, volumes were split by eye.

### OCT image analysis using DL algorithms

In most cases, transplants were not consistently aligned with either the longitudinal or transverse OCT scans; therefore, 2 OCT B-scans were exported from each of both the OCT volumes per transplant per time point (4 total B-scans); scans at the one-third and two-thirds marks between the first and last occurrence of the transplant in the OCT volume were used for analysis. Corresponding baseline images at the same location were also used for analysis.

All exported TIF files were cropped to isolate the B-scan of interest by removing the corresponding fundus image and metadata. OCT scans recorded during baseline evaluations were segmented by U-Net #1. Scans recorded after laser or transplant procedures were segmented by either U-Net #2 or a combination of U-Net #2 and #3. All segmentation was manually evaluated and corrected, if necessary, to ensure accurate analysis. Subsequently, segmentation maps representing the pixel-wise delineation of retinal layers were generated, allowing for visual representation of the segmentation results. PLGA-iRPE, PLGA-only, and lasered regions were isolated (if applicable) within each resultant segmentation map. The total area (in pixels) of NFL, GCL/IPL, INL, and ONL above transplants and in lasered regions was calculated for each image using custom MATLAB scripts. The area of the NFL, GCL/IPL, INL, and ONL layers was normalized to the respective baseline image and averaged across all 4 scans.

### OCT-A image analysis

OCT-A scans were exported from the HEYEX 2 software in the TIF format. Choriocapillaris masks were generated for each ROI within the scan: healthy, laser, PLGA-iRPE, and PLGA-only. These masks were used to segment the OCT-A signal and calculate the gray value, or the average binary pixel intensity, of the choriocapillaris for each ROI. All gray value calculations were completed using ImageJ (NIH). For each image, the gray value of laser, PLGA-iRPE transplant, and PLGA-only transplant regions was normalized to the healthy region within the same image and was reported as a percentage change. Normalization of the data to each image’s healthy region minimized the technical and biological differences between animals and image acquisition.

### Immunostaining and imaging

Tissue sections were deparaffinized in xylene, followed by rehydration in a graded series of ethanol solutions, and washed with water. Antigen retrieval of 4% paraformaldehyde-fixed eyes was achieved by heating the sections in Universal HIER Antigen Retrieval Buffer (AB208572; Abcam) for 20 minutes at 95°C. Following antigen retrieval, tissue sections were then blocked with 2% normal donkey serum in TBS-Tween (TBST) for 1 hour and incubated overnight at room temperature in the following primary antibodies: anti-Ku80 (MA5-14953; Thermo Fisher Scientific) and anti-PMEL17 (M0634; Dako, Agilent) at 1:100 dilution in TBST containing 2% normal donkey serum. Following primary antibody labeling, sections were washed in TBST and incubated for 2 hours at room temperature with a donkey anti-rabbit antibody conjugated with Alexa Fluor 555 (1:500; A31572, Thermo Fisher Scientific) or Alexa Fluor 647 (1:500; A32795; Thermo Fisher Scientific) and either one of the following antibodies: Alexa Fluor 647–conjugated isolectin Griffonia simplifolica IB4 (1:500; I32450; Thermo Fisher Scientific) or Cy3-labeled PNA (1:200; CL-1073-NB, Novus Biologicals, Bio-Techne). Nuclei were counterstained with DAPI (1:2,000; 62248; Thermo Fisher Scientific). Images were captured at 25× original magnification using an LSM 980 (ZEISS) with an Airyscan 2 confocal microscope (ZEISS).

### Statistics

Statistical analyses and plot generations were performed in Prism (GraphPad Software) and R Studio. All data were first assessed for normality and were thus treated as non-normal.

A mixed effects model using REML was applied to both the AO and OCT-A data. For post hoc analysis, Tukey’s multiple comparisons test was used for AO data, while Bonferroni’s correction was applied to OCT-A data to evaluate the effects of measurement time points on the specified metric. For OCT data, the Friedman test was used to assess repeated measures data, while the Dunn test was employed to compare groups at each time point. Box plot graphs were created to represent the data; each box extended from minimum to maximum value; the 25th percentile, 75th percentile, and median were represented; and all data points were plotted. Only the significant *P* values were plotted. *P* values of less than 0.05 were considered significant.

### Study approval

All animal procedures were performed in accordance with the guidelines of the Association for Research in Vision and Ophthalmology statement for the use of animals in ophthalmic and vision research and received prior approval from the NIH institute Animal Care & Use Committee.

### Data availability

Original images are provided in the main and supplemental figures, and raw data values for quantitative analysis are provided in supplemental tables and the Supporting Data Values file.

## Author contributions

Rohan Gupta, IB, Rishabh Gupta, DB, JA, FB, RS, HQ, AM, MTMDQC, MSS, and AHK conducted experiments. Rohan Gupta, IB, BA, CGC, JA, ED, RS, AM, MTMDQC, AHK, and KB contributed to study design, data analysis, and manuscript writing. KB approved the manuscript.

## Supplementary Material

Supplemental data

Supplemental video 1

Supporting data values

## Figures and Tables

**Table 1:**
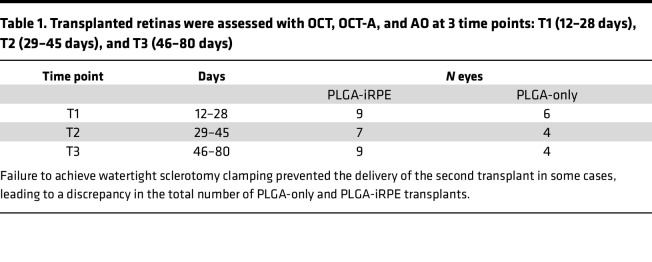
Transplanted retinas were assessed with OCT, OCT-A, and AO at 3 time points: T1 (12–28 days), T2 (29–45 days), and T3 (46–80 days)

## Version 1 05/22/2025

Electronic publication

## References

[1] Wong WL (2014). Global prevalence of age-related macular degeneration and disease burden projection for 2020 and 2040: a systematic review and meta-analysis. Lancet Glob Health.

[2] Hanany M (2020). Worldwide carrier frequency and genetic prevalence of autosomal recessive inherited retinal diseases. Proc Natl Acad Sci U S A.

[3] Botto C (2022). Early and late stage gene therapy interventions for inherited retinal degenerations. Prog Retin Eye Res.

[4] Miotti G (2021). Stem cell therapy in ocular pathologies in the past 20 years. World J Stem Cells.

[5] Padhy SK (2020). Voretigene neparvovec and gene therapy for Leber’s congenital amaurosis: review of evidence to date. Appl Clin Genet.

[6] Borchert GA (2023). The role of inflammation in age-related macular degeneration-therapeutic landscapes in geographic atrophy. Cells.

[7] Akiba R (2023). Progress of iPS cell-based transplantation therapy for retinal diseases. Jpn J Ophthalmol.

[8] Khateb S (2021). Cell-based therapies for age-related macular degeneration. Adv Exp Med Biol.

[9] Bose D (2023). Considerations for developing an autologous induced pluripotent stem cell (iPSC)-derived retinal pigment epithelium (RPE) replacement therapy. Cold Spring Harb Perspect Med.

[10] Sharma R (2019). Clinical-grade stem cell-derived retinal pigment epithelium patch rescues retinal degeneration in rodents and pigs. Sci Transl Med.

[11] Sharma R (2020). Retinal pigment epithelium replacement therapy for age-related macular degeneration: are we there yet?. Annu Rev Pharmacol Toxicol.

[12] May-Simera HL (2018). Primary cilium-mediated retinal pigment epithelium maturation is disrupted in ciliopathy patient cells. Cell Rep.

[13] Kashani AH (2020). Surgical method for implantation of a biosynthetic retinal pigment epithelium monolayer for geographic atrophy: experience from a phase 1/2a study. Ophthalmol Retina.

[14] Carpenter CL (2018). Normative retinal thicknesses in common animal models of eye disease using spectral domain optical coherence tomography. Adv Exp Med Biol.

[15] Koss MJ (2016). Subretinal implantation of a monolayer of human embryonic stem cell-derived retinal pigment epithelium: a feasibility and safety study in Yucatán minipigs. Graefes Arch Clin Exp Ophthalmol.

[16] Sanchez I (2011). The parameters of the porcine eyeball. Graefes Arch Clin Exp Ophthalmol.

[17] Oliveira C (2007). Axial length and optic disc size in normal eyes. Br J Ophthalmol.

[18] Scharf J (2021). Optical coherence tomography angiography of the choriocapillaris in age-related macular degeneration. J Clin Med.

[19] Barone F (2023). A versatile laser-induced porcine model of outer retinal and choroidal degeneration for preclinical testing. JCI Insight.

[20] Zou KH (2004). Statistical validation of image segmentation quality based on a spatial overlap index. Acad Radiol.

[21] Yeghiazaryan V, Voiculescu I (2018). Family of boundary overlap metrics for the evaluation of medical image segmentation. J Med Imaging (Bellingham).

[22] Huckenpahler AL (2019). Noninvasive imaging and correlative histology of cone photoreceptor structure in the pig retina. Transl Vis Sci Technol.

[23] Bharti K (2006). The other pigment cell: specification and development of the pigmented epithelium of the vertebrate eye. Pigment Cell Res.

[24] Cibulskyte D (2005). Chronic cyclosporine nephrotoxicity: a pig model. Transplant Proc.

[25] Kugelman J (2020). Retinal boundary segmentation in Stargardt disease optical coherence tomography images using automated deep learning. Transl Vis Sci Technol.

[26] Ortolan D (2022). Single-cell-resolution map of human retinal pigment epithelium helps discover subpopulations with differential disease sensitivity. Proc Natl Acad Sci U S A.

[27] Kostic C, Arsenijevic Y (2016). Animal modelling for inherited central vision loss. J Pathol.

