# Supplementary material for: iPSC-RPE patch restores photoreceptors and regenerates choriocapillaris in a pig retinal degeneration model
Source: JCI Insight. 2025 May 22;10(10):e179246. doi: 10.1172/jci.insight.179246 (PMC12128979; doi:10.1172/jci.insight.179246)
Supplement: Supplemental data [file jciinsight-10-179246-s031.pdf]

SUPPLEMENTARY FIGURES

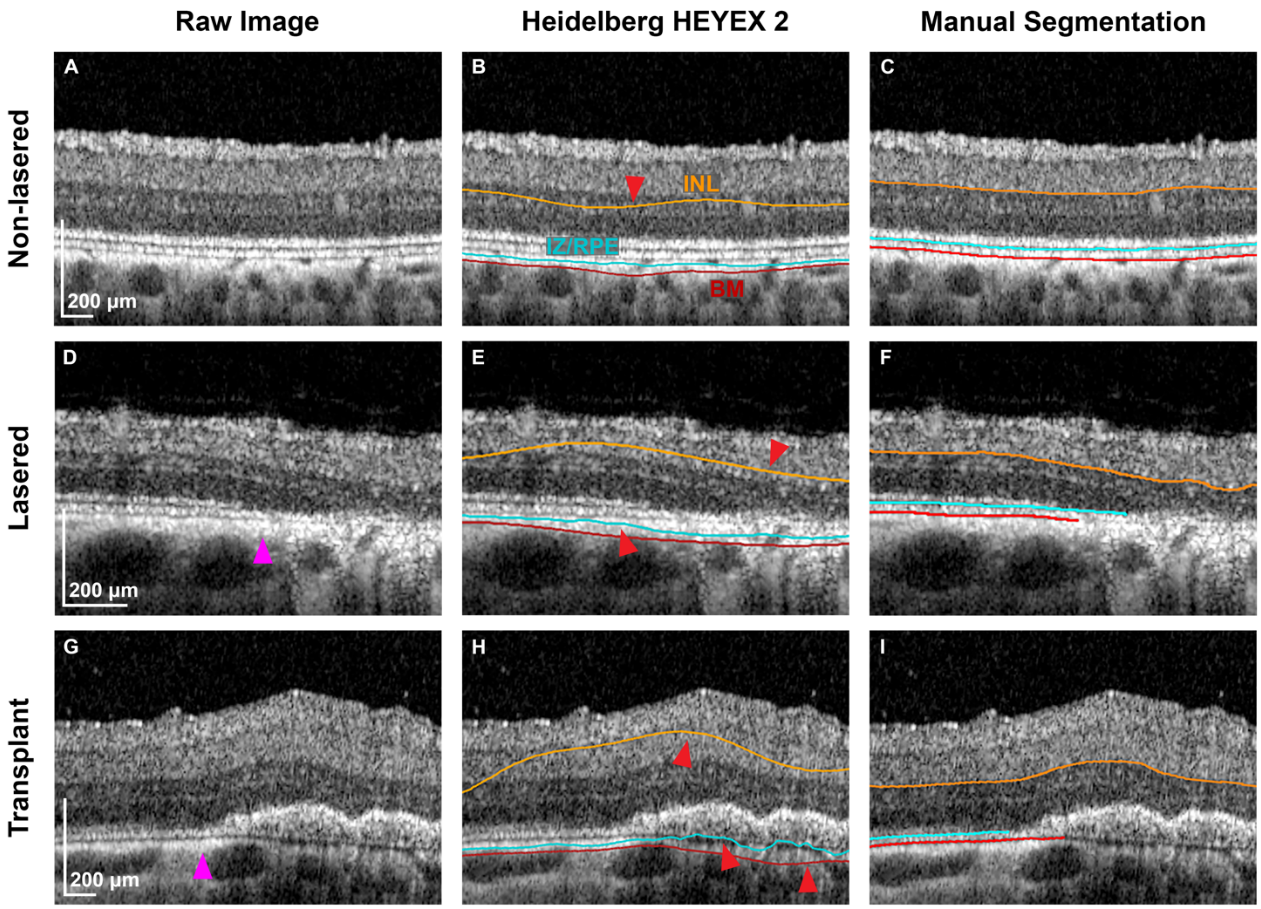

Supplemental Figure 1. Comparison of manual and automatic segmentation generated by Heidelberg HEYEX 2 software in magnified OCT B-scans. The annotations for the inner aspect of the inner nuclear layer (INL), interdigitation zone/retinal pigment epithelium (IZ/RPE), and Bruch's membrane (BM) are highlighted in orange, cyan, and red, respectively. **(A-C)** Segmentation comparisons in non-lasered or untreated regions. **(D-F)** Segmentation comparisons on the boundary of lasered regions; the pink arrow defines the boundary between untreated (left) and the lasered (right) areas. **(G-I)** Segmentation comparisons on the boundary of PLGA-iRPE transplant at T2; the pink arrowhead defines the boundary between untreated (left) and the transplant (right) areas. Red arrowheads point to errors in segmentation.

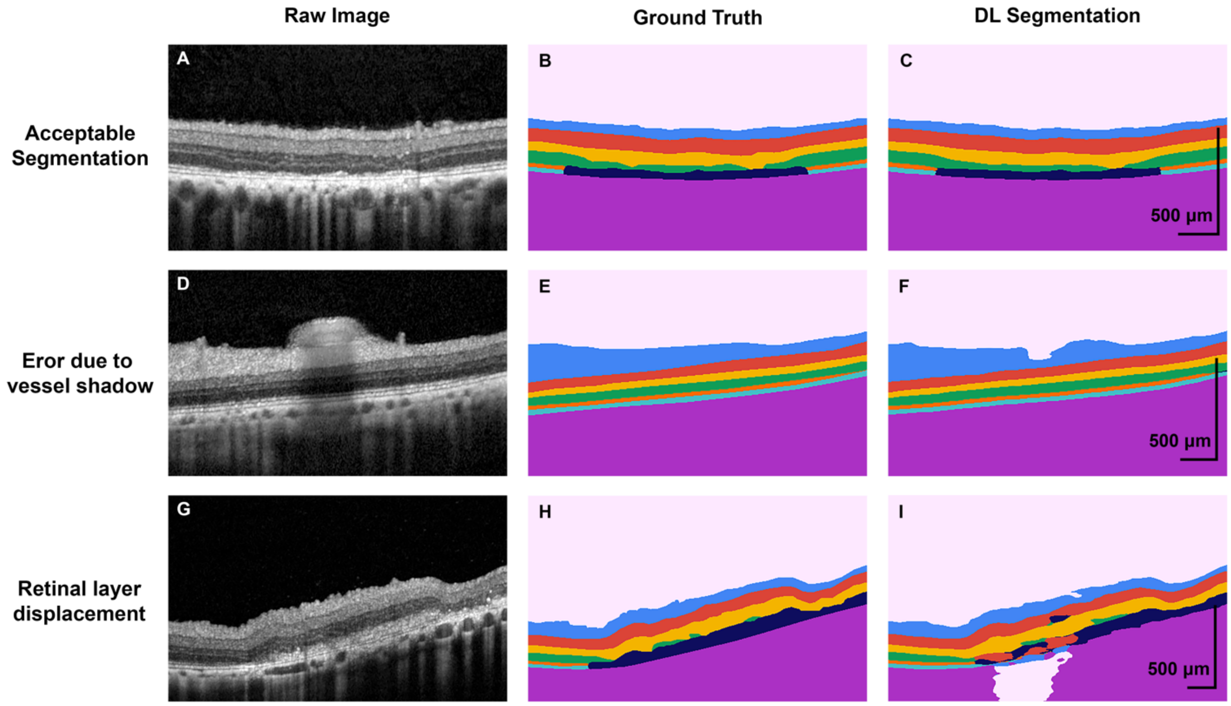

Supplemental Figure 2. Representative retinal layer and damaged zone (DZ) segmentation generated by U-Net #2 on cropped and magnified OCT B-scans. (A, D, G) Raw OCT B-scans. **(B, E, H)** Corresponding ground truth segmentation maps generated by expert graders delineating retinal layers and DZ. **(C, F, I)** Segmentation map generated by U-Net #2 delineating retinal layers and DZ. **(A-C)** Example of an instance where the predicted segmentation was deemed acceptable by expert graders. **(D-F)** Example of an instance where nerve fiber layer segmentation was compromised due to vessel shadow. **(G-I)** Example of an instance where retinal layers were displaced axially due to unusually hyporeflective choroidal blood vessels.

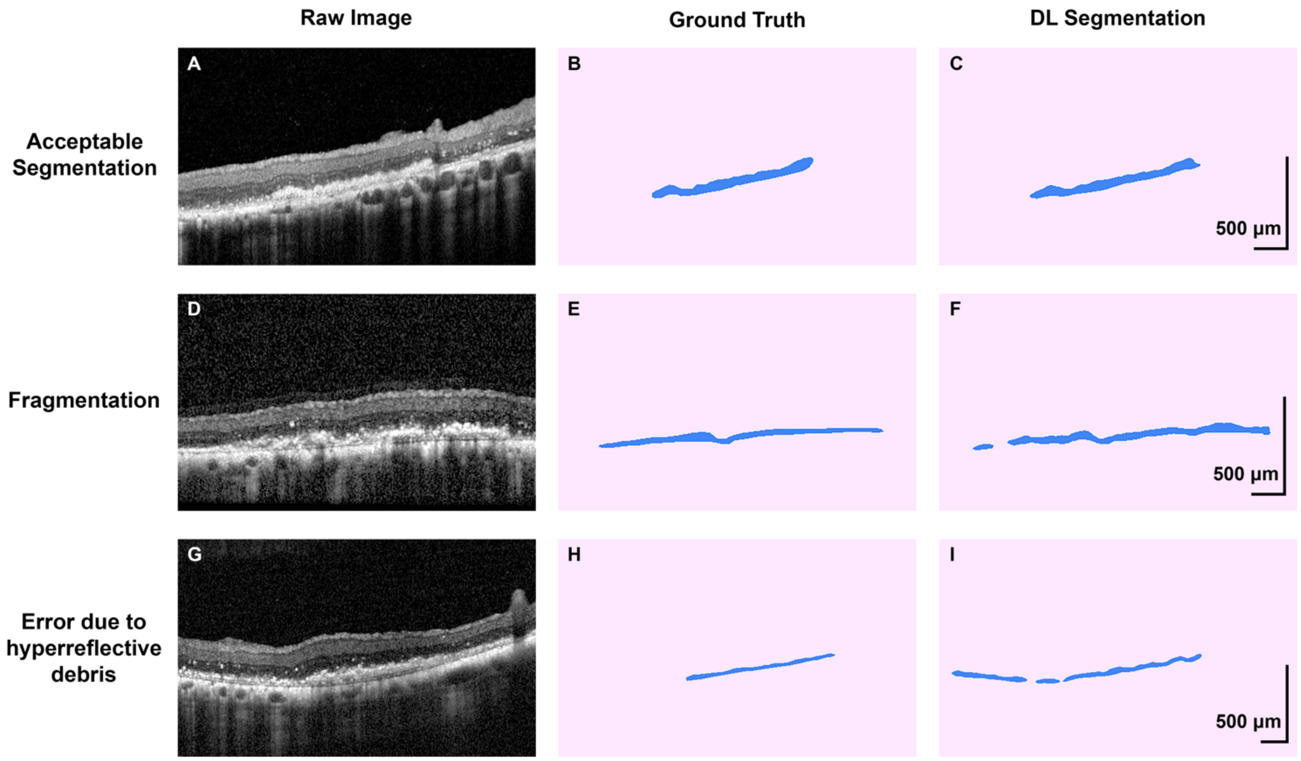

Supplemental Figure 3. Representative transplant segmentation generated by U-Net #3 on cropped and magnified OCT B-scans. (A, D, G) Raw OCT B-scans. **(B, E, H)** Corresponding binary ground truth segmentation maps generated by expert graders delineating the transplant. **(C, F, I)** Binary segmentation map generated by U-Net #3 delineating the transplants. **(A-C)** Example of an instance where the predicted segmentation was deemed acceptable by expert graders. **(D-F)** Example of an instance where transplant segmentation was fragmented. **(G-I)** Example of an instance where outer retinal hyperreflective debris from laser injury resulted in a falsely expanded transplant segmentation.

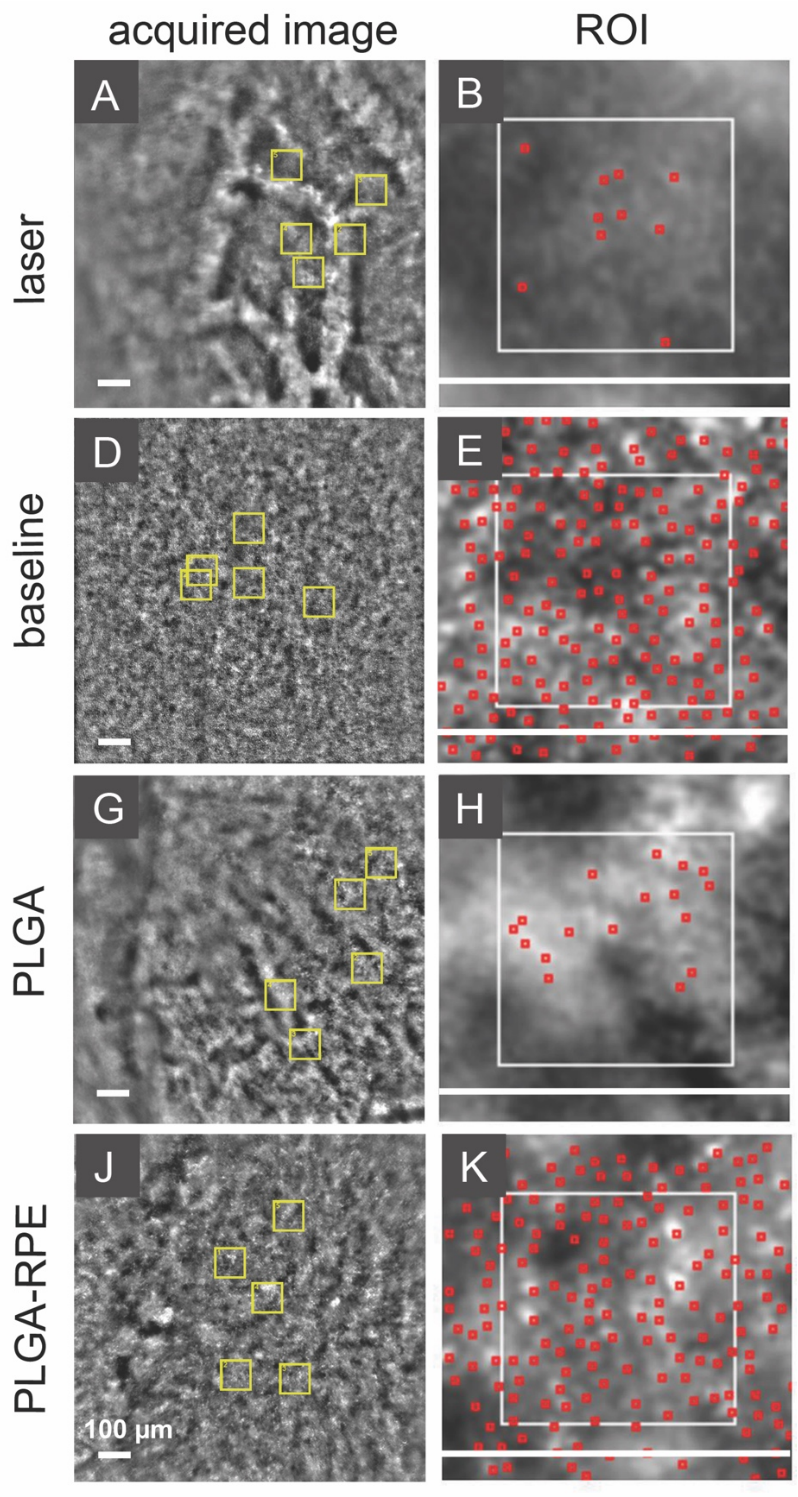

Supplementary Figure 4. **Adaptive optics segmentation and analysis**. For each eye, three images were acquired per condition: lasered retina **(A)**, baseline (**D**, only for day -7), PLGA-only **(G)**, and PLGA-iRPE **(J)** at each time point T1, T2, and T3. For each image the photoreceptors in five randomly selected regions of interest (ROI) were identified using the software provided with the equipment and the number of segmentable photoreceptor loci were counted for each condition: lasered retina **(B)**, baseline **(E)**, PLGA-only **(H)**, and PLGA-iRPE **(K)**.

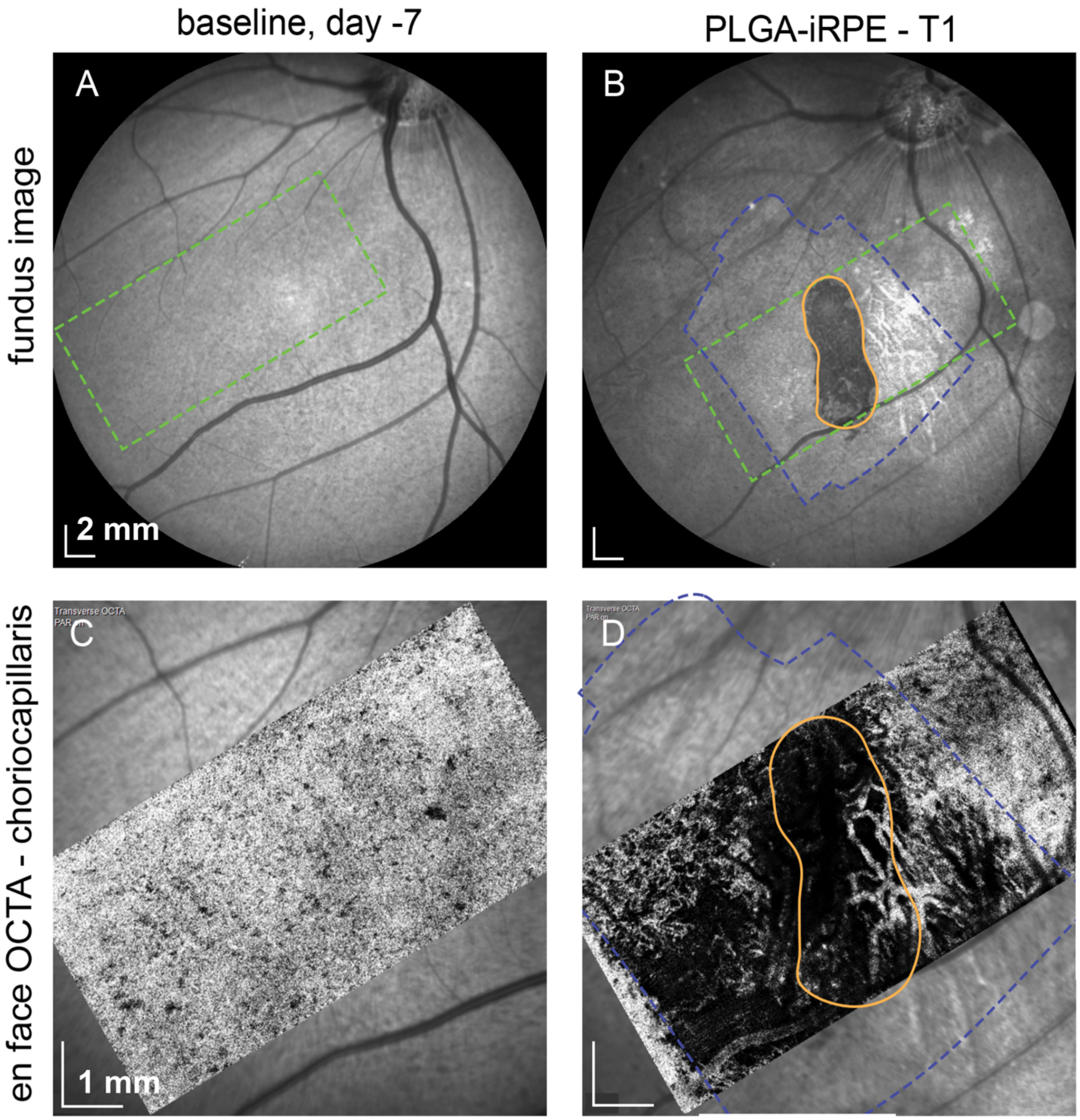

Supplementary Figure 5. PLGA-iRPE transplant supports minimal choriocapillaris regeneration at T1 time point. (A-D) Representative fundus **(A, B)** and OCT-A **(C, D)** images of baseline examination **(A, C)** and at follow-up time point T1 after PLGA-iRPE transplant **(B, D)**.

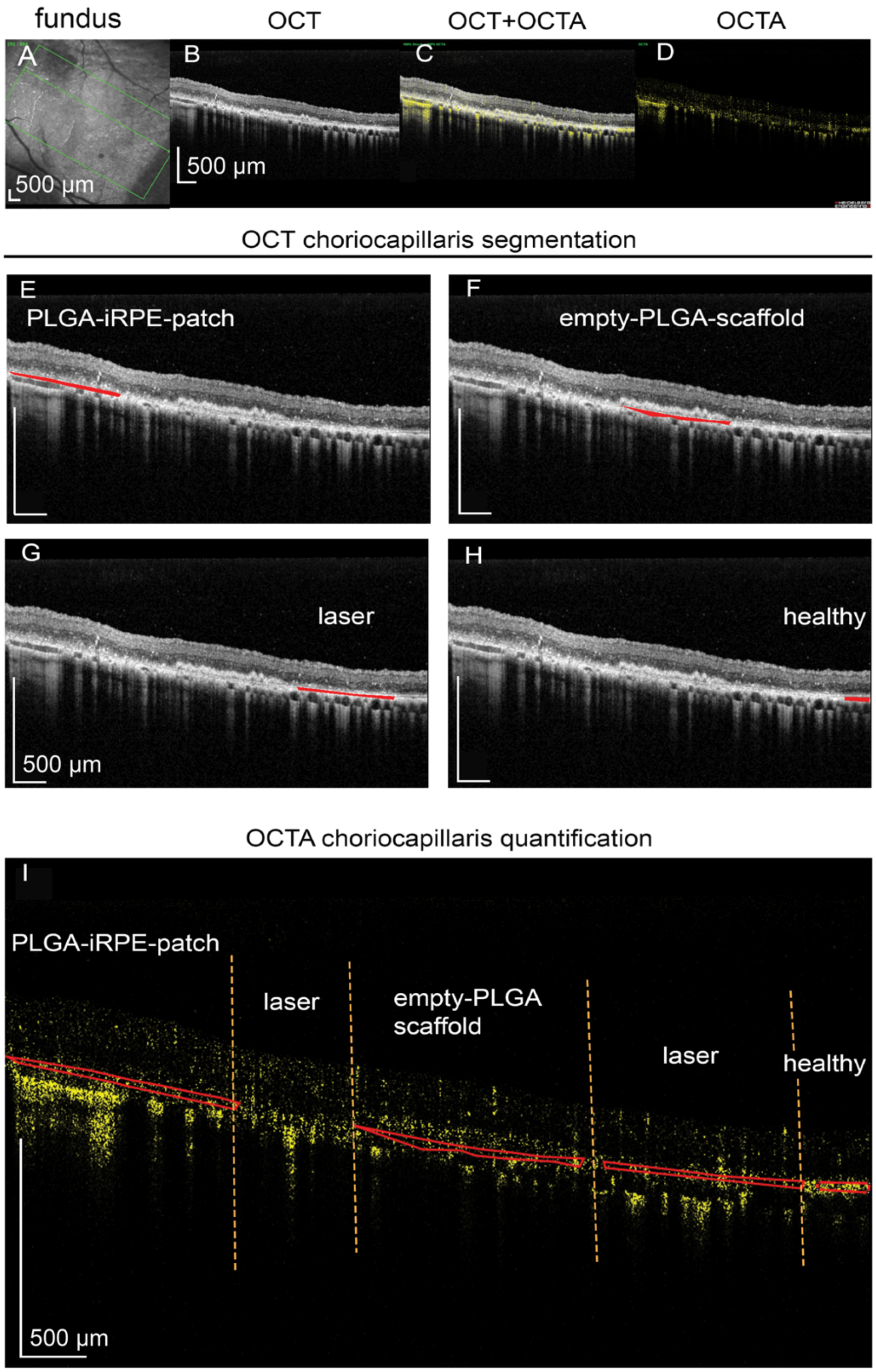

Supplementary Figure 6. **OCT-A segmentation and choriocapillaris quantification protocol**. **(A-D)** Three scan images were downloaded for each treated eye. Each image is composed by an infrared fundus image showing the location of the scan **(A)**, an OCT image of corresponding to the location **(B)**, an image of the vessel signals overlayed to the OCT scan **(C)**, and the isolated vessels signal represented in yellow **(D)**. **(E-H)** Using the OCT scan shown in **(B)** a region of interest (red line) was created to segment choriocapillaris for: PLGA-iRPE **(E)**, PLGA-only **(F)**, laser-injured retina **(G)** and healthy retina **(H)**. **(I)** Each region of interest (red line) was then overlaid to the isolated vessels signal to calculate the gray pixel value. Data were normalized to the healthy area for the analysis.

SUPPLEMENTARY TABLES

Dice Coefficient																				
	Vitreous		NFL		GCL/IPL		INL		ONL		EZ		IZ/RPE		Choroid/Sclera		DZ		Macro	
	Mean	SD	Mean	SD	Mean	SD	Mean	SD	Mean	SD	Mean	SD	Mean	SD	Mean	SD	Mean	SD	Mean	SD
Exp 1	0.9957	0.0068	0.9312	0.0202	0.9496	0.0156	0.8927	0.0457	0.8986	0.0463	0.8417	0.0581	0.8663	0.0553	0.9926	0.0094	0.8120	0.0820	0.9089	0.0241
Exp 2	0.9976	0.0041	0.9309	0.0204	0.9523	0.0158	0.8932	0.0506	0.8988	0.0548	0.8492	0.0546	0.8666	0.0574	0.9958	0.0065	0.8010	0.0924	0.9095	0.0241
Exp 3	0.9925	0.0146	0.9212	0.0360	0.9508	0.0216	0.8940	0.0502	0.9026	0.0504	0.8304	0.0689	0.8637	0.0537	0.9863	0.0257	0.7893	0.0919	0.9034	0.0291
Exp 4	0.9982	0.0015	0.9219	0.0226	0.9517	0.0179	0.8964	0.0542	0.9031	0.0468	0.8351	0.0557	0.8547	0.0586	0.9966	0.0020	0.8081	0.0799	0.9073	0.0247

Average Surface Distance (pixels)																
	NFL		GCL/IPL		INL		ONL		EZ		IZ/RPE		Choroid/Sclera		DZ	
	Mean	SD	Mean	SD	Mean	SD	Mean	SD	Mean	SD	Mean	SD	Mean	SD	Mean	SD
Exp 1	0.675	0.250	2.044	0.627	1.902	1.777	4.212	9.460	2.445	4.347	2.348	3.329	2.096	2.388	8.491	14.278
Exp 2	0.732	0.426	0.743	0.303	2.200	2.634	5.040	11.219	7.039	25.875	8.848	27.627	0.554	0.785	5.189	7.088
Exp 3	0.967	1.348	0.844	0.779	1.961	1.994	5.886	16.699	1.972	2.320	2.231	2.912	1.877	2.509	6.659	9.101
Exp 4	0.819	0.419	0.753	0.276	1.948	2.119	4.943	12.979	2.701	4.368	7.282	15.320	1.311	1.648	6.609	9.765

Hausdorff Distance 95% (pixels)																
	NFL		GCL/IPL		INL		ONL		EZ		IZ/RPE		Choroid/Sclera		DZ	
	Mean	SD	Mean	SD	Mean	SD	Mean	SD	Mean	SD	Mean	SD	Mean	SD	Mean	SD
Exp 1	2.6066	4.6029	21.9472	7.0693	16.6968	25.6332	38.5430	91.6413	29.6232	96.5668	25.8573	43.7148	23.5062	54.8354	69.8823	113.4886
Exp 2	3.0063	6.1911	2.5382	3.2399	22.1484	33.6444	46.1003	97.0537	58.5698	178.8048	76.4940	205.0077	3.3265	7.3259	40.2913	65.8279
Exp 3	5.3724	14.4013	3.7298	8.7160	19.6361	29.2302	47.2089	120.4463	20.7645	32.4411	23.9058	37.2313	17.2434	28.7112	57.0544	91.4853
Exp 4	3.0538	2.5996	2.4700	1.5514	18.6489	28.4812	45.4072	108.6725	35.3988	100.0023	92.7965	197.3430	13.2711	21.6251	58.3439	92.5913

Supplementary Table 1. Raw data for all U-Net #2 experiments. Each experiment evaluates a deep learning architecture with unique parameters. The highest-performing model, identified through qualitative and quantitative assessment, was used for OCT analysis. The trained model from experiment 2 was selected. The mean and standard deviations for dice coefficients are provided for all retinal layers, vitreous, choroid/sclera, and the damaged zone, along with the macro dice coefficient. Mean and standard deviation for surface distance (pixels) and Hausdorff Distance (95%) (pixels) are also reported for each retinal layer and the damaged zone.

	Macro Dice Coefficient		Transplant Dice Coefficient		Average Surface Distance		Hausdorff Distance 95%	
	Mean	SD	Mean	SD	Mean	SD	Mean	SD
Exp 1	0.8556	0.0767	0.7123	0.1529	6.2547	11.4213	41.7241	61.2849
Exp 2	0.8785	0.0583	0.7580	0.1162	5.6272	8.8893	44.9184	61.6063
Exp 3	0.8428	0.1006	0.6868	0.2005	11.6000	21.5267	68.3034	109.6780
Exp 4	0.8549	0.0660	0.7109	0.1314	10.3889	12.4152	93.8323	124.5437
Exp 5	0.7987	0.1216	0.5987	0.2425	10.6668	15.7811	60.9862	81.0529

Supplementary Table 2. Raw data for all U-Net #3 experiments. Each experiment evaluates a deep learning architecture with unique parameters. The highest-performing model, identified through qualitative and quantitative assessment, was used for OCT analysis. The trained model from experiment 2 was selected. The mean and standard deviation of the transplant and macro dice coefficient are provided. Mean and standard deviation of the average surface distance (pixels) and Hausdorff Distance (95%) (pixels) are also reported.

	B			T1 (14-28 days)			T2 (29-45 days)			T3 (46-80 days)		
	Mean	SD	N	Mean	SD	N	Mean	SD	N	Mean	SD	N
untreated	21344.5	1970.683	6									
PLGA-iRPE				16993.27	958.9036	4	17770.67	711.141	4	17556.34	809.3274	6
PLGA-only				9357.622	6106.295	4	5456.273	7473.663	4	7569.562	5036.823	4
laser				1047.092	394.7626	6	1047.092	394.7626	6	1188.151	527.404	6

Supplementary Table 3. Raw value of cone photoreceptors cell density (cells/mm^2) assessed by AO. Data represents the number of cells/mm^2 and is reported as mean, standard deviation (SD), and sample size (N).

	T1 (14-28 days)			T2 (29-45 days)			T3 (46-80 days)		
	Mean	SD	N	Mean	SD	N	Mean	SD	N
PLGA-RPE	50.4054	46.13045	5	10.66133	18.46597	3	35.9196	52.53568	5
PLGA	66.5978	37.66403	5	59.321	53.33935	3	39.3438	36.00358	5
laser	131.1798	51.36943	5	83.82867	27.30252	3	121.257	38.56843	5
healthy	265.2172	37.77592	5	187.0277	126.9738	3	187.79	36.21677	5

Supplementary Table 4. Raw pixel values of choriocapillaris quantification assessed by OCT-A. Data is reported as mean, standard deviation (SD), and sample size (N).
